# Insights into the Activation Mechanism of HCA1, HCA2, and HCA3

**DOI:** 10.1021/acs.jmedchem.4c02567

**Published:** 2025-02-12

**Authors:** Jiening Wang, Yuxia Qian, Zhen Han, Yize Wang, Yanru Liu, Jie Li, Qingmiao Duanmu, Sheng Ye, Anna Qiao, Shan Wu

**Affiliations:** † State Key Laboratory of Biocatalysis and Enzyme Engineering, Hubei Collaborative Innovation Center for Green Transformation of Bio-Resources, Hubei Key Laboratory of Industrial Biotechnology, School of Life Sciences, Hubei University, Wuhan, Hubei 430062, China; ‡ Tianjin Key Laboratory of Function and Application of Biological Macromolecular Structures, School of Life Sciences, Tianjin University, 92 Weijin Road, Nankai District, Tianjin 300072, China

## Abstract

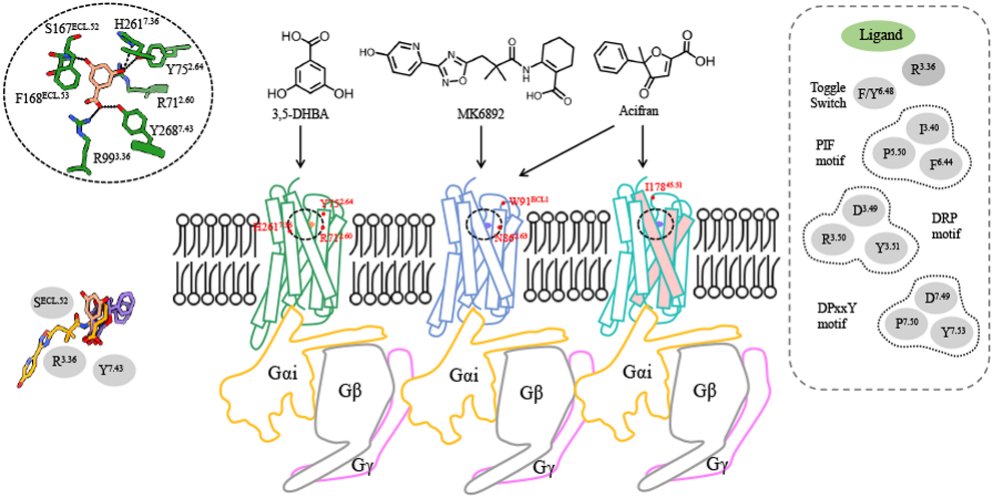

Hydroxy-carboxylic acid receptors HCA1, HCA2, and HCA3 can be activated by important intermediates of energy metabolism. Despite the research focusing on HCA2, its clinical application has been limited by adverse effects. Therefore, the role of HCA1 as a promising target for the treatment of lipolysis warrants further exploration. As HCAs exhibit high similarity when activated with diverse selective agonists, a conserved yet unique activation mechanism for HCAs remains undisclosed. Herein, we unveil the cryo-electron microscopy structures of the 3,5-DHBA-HCA1-Gi signaling complex, the acifran- and MK6892-bound HCA2-Gi signaling complexes, and the acifran-HCA3-Gi signaling complex. Comparative analysis across HCAs reveals key residues in HCA1 contributing to the stabilization of the ligand-binding pocket. Furthermore, chimeric complexes and mutational analyses identify residues that are pivotal for HCA2 and HCA3 selectivity. Our findings elucidate critical structural insights into the mechanisms of ligand recognition and activation within HCA1 and broaden our comprehension of ligand specificity binding across the HCA family.

## Introduction

Hydroxy-carboxylic acids possess both hydroxyl and carboxyl functional groups, playing a pivotal role as intermediates in energy metabolism across various biological processes, such as lipolysis, tumor proliferation, and inflammation.^1−3^ These compounds have been identified as the physiological ligands for a family of G-protein-coupled receptors designated as HCA receptors, which consist of three members: HCA1, HCA2, and HCA3.^1,2,4,5^ Upon activation, these receptors engage with Gi protein, leading to a reduction in cyclic AMP (cAMP) and adenylate cyclase activity.^4^

HCA1, also referred to as GPR81, exhibits pronounced expression in adipose tissue and to a lesser extent in the kidney, skeletal muscle, and liver,^6−8^ with lactate serving as its endogenous agonist.^9^ During physical exercise or under hypoxic conditions, lactate level may elevate sufficiently to selectively activate HCA1 without affecting HCA2 and HCA3.^9,10^ Activation of HCA1 occurs through both autocrine and paracrine mechanisms, aiding in the modulation of antilipolytic effects and the attenuation of weight gain.^11−13^ This activation mechanism presents a potential therapeutic avenue for dyslipidemia, offering advantages in terms of reduced side effects compared with HCA2.^14^ Moreover, HCA1 is crucial for tumor metabolism and tumor immunity, probably due to the reduction of adipocyte metabolism.^11,15^ HCA1 expression in microglia and astrocytes has been shown to diminish NLRP3 inflammasome assembly, thereby curtailing innate immune cell activity.^16−18^ Given that HCA1 knockout results in increased cancer cell mortality, HCA1 emerges as a promising therapeutic target for cancer treatment.^11,19−21^

Several agonists for HCA1 have been discovered, including 3-HBA, 3,5-DHBA, and 3Cl-5OH-BA, despite the presence of the endogenous ligand lactate (Figure S2a). Notably, 3,5-DHBA and 3Cl-5OH-BA act as selective agonists for HCA1, while 3-HBA can activate both HCA1 and HCA2.^22^ The distinguishing feature between 3-HBA and 3,5-DHBA lies in the presence of an additional hydroxyl group. In addition, compounds targeting HCA1, such as AZ2, and compound 2 have shown promise in treating dyslipidemia and cardiovascular control^14,22−24^ (Figure S2a). Meanwhile, peptide 7w-2 has been investigated due to its ability to inhibit lipolysis and significantly suppress tumor growth.^11^ However, no antagonist for HCA1 has been identified thus far. A sequence identity of 52% between HCA1 and HCA2 is noted, with significant similarity observed between HCA2 and HCA3, indicating subtle structural and ligand selectivity differences. β-Hydroxybutyrate, differing from lactate by merely the site of a hydroxyl group and an extra methylene group, serves as the endogenous agonist for HCA2 and exhibits low affinity for HCA3, showing no activity toward HCA1^1^ (Figure S2b). Activation of HCA2 is associated with antilipolytic and anti-inflammatory effects, positioning it as a viable target for hyperlipidemias and inflammation-related diseases.^2^ As severe flushing would be caused in clinical trials by the β-arrestin signaling pathway of HCA2, synthetic agonists, such as MK6892, acipimox, and N2L, have been developed and MK6892 is also identified as a selective agonist of HCA2,^25−27^ while acifran demonstrates comparable affinity toward both HCA2 and HCA3 (Figure S2b). Despite the high sequence homology between HCA2 and HCA3, 2- and 3-hydroxylated-medium-chain fatty acids, particularly 3-hydroxyocanoate acid, as well as D-amino acids, represent specific agonists for HCA3 (Figure S2c). According to the chemical structure of agonists of HCAs, the conserved hydroxyl and carboxyl represent the conserved functional groups in the ligands for HCAs. The ligands of HCA1 generally have shorter main chain lengths, except for the compounds of HCA1 and HCA2, which may perform in the allosteric pathway (Figure S2).

To date, despite extensive investigation into the architectures of HCA2, including its inactive form, agonist engagement, and biased allosteric activation, the structural elucidation of HCA1 remains unaccomplished.^28−37^ To unveil the ligand-binding mode and signal transduction mechanisms of HCA1, we have resolved the cryo-electron microscopy (cryo-EM) structure of human 3,5-DHBA-bound HCA1 at a resolution of 2.70 Å. Additionally, to deepen our comprehension of the molecular mechanisms by which agonists modulate and activate the respective HCAs, we have determined the structures of MK6892-bound HCA2, acifran-bound HCA2, and acifran-bound HCA3, each in complex with the heterotrimeric Gi protein at resolutions of 2.80, 2.73, and 3.01 Å, respectively. Coupled with mutagenesis studies, our research sheds light on both the shared and distinct agonist binding models across HCAs and the mechanisms underpinning G-protein activation.

## Results

### Overall Structures of HCAs-Gi Complex with Diverse Agonists

**Figure 1:**
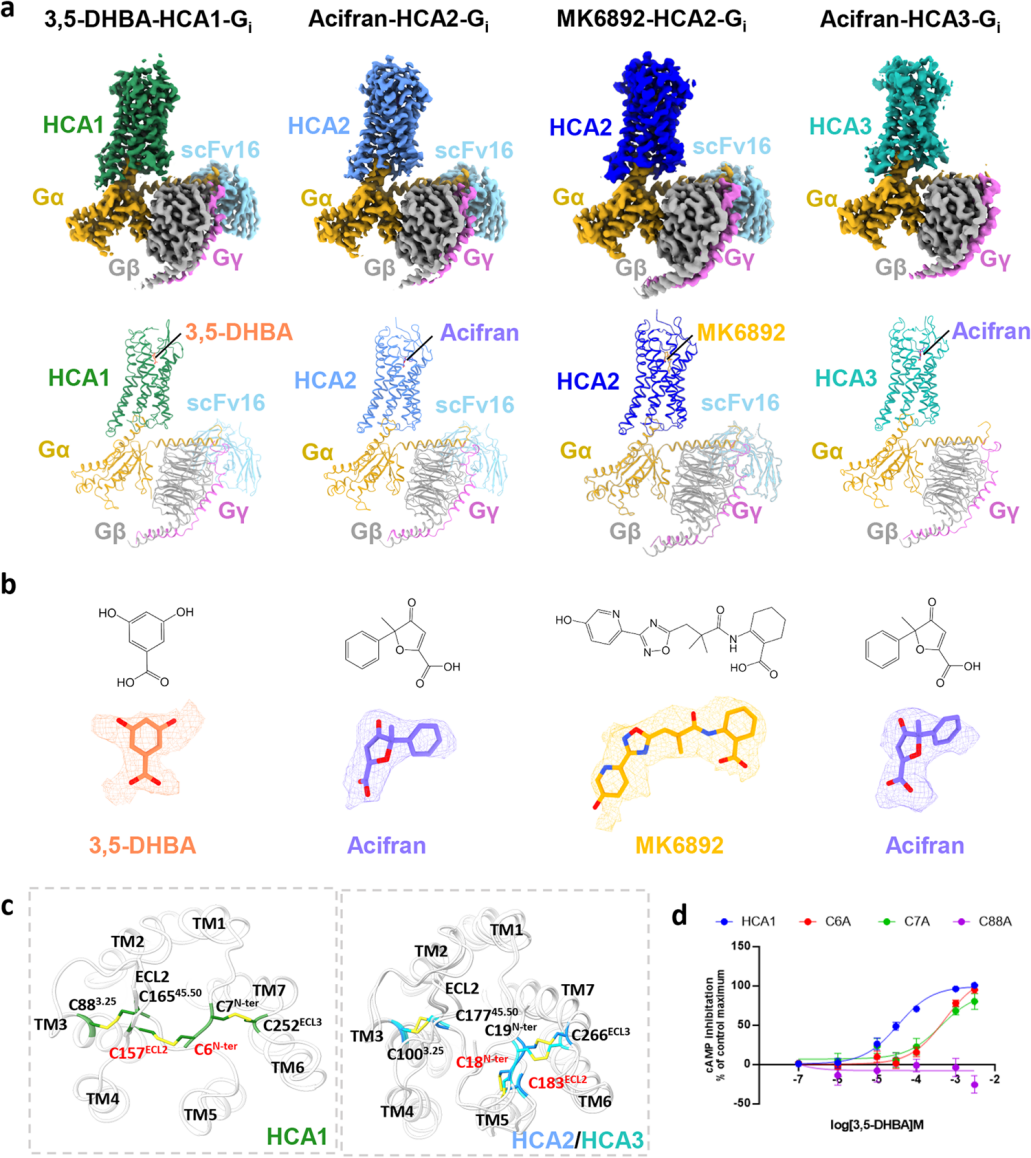
Overall structure of the complex of HCA1, HCA2, and HCA3. (a) Cryo-EM density maps and the ribbon structures of 3,5-DHBA-bound HCA1 (PDB: 9KT9), acifran- (PDB: 9KT8), MK6892-bound HCA2 (PDB: 9KT7), and acifran-bound HCA3 (PDB: 9KT6). Color schemes are indicated by the labels. (b) The chemical structure and the stick representation of ligands of HCAs (3,5-DHBA, acifran, and MK6892), with cryo-EM densities shown in the mesh mode. 3,5-DHBA is depicted in salmon, acifran in medium slate blue, and MK6892 in goldenrod. (c) The compact conformation of the extracellular side of HCAs, represented with three pairs of disulfide bonds. Six cystines were shown in stick representation. (d) Represented curve for 3,5-DHBA-induced activation of HCA1 mutations of cystines examined by the cAMP inhibition assay. Data are presented as means ± standard error of the mean (SEM) from three independent experiments, which were performed in triplicate.

To delineate the molecular mechanisms of HCA1 functionality, we separately purified human HCA1/HCA2/HCA3, Gαs, His6-tagged Gβ1, and Gγ2, subsequently coexpressing them to infect *Spodoptera frugiperda* cells. Following resuspension, the cells underwent incubation with IBA resin and subsequent washing with an identical buffer containing various agonists. To stabilize the HCA1/HCA2-Gi complex, the single-chain antibody scFv16 was additionally incorporated into the HCA1 and HCA2 complex. The ensuing protein complex was concentrated and purified via a Superdex 200 Increase 10/300 column. The structures of 3,5-DHBA-bound HCA1, MK6892-bound HCA2, acifran-bound HCA2, and acifran-bound HCA3 in conjunction with the Gi protein heterotrimer were ascertained to overall resolution of 2.70, 2.80, 2.73, and 3.01 Å, respectively (Figures 1a, S3, and S4). High-resolution density maps facilitated precise modeling of the majority of residues within the seven transmembrane (TM) domains, extracellular loop 1–3 (ECL1–3), and intracellular loop 1 and 3 (ICL1/ICL3) across the trio of HCAs, as well as scFv16 in HCA1 and HCA2. However, the density associated with intracellular loop 2 (ICL2) and the C-terminal regions of the receptors was comparatively weak, suggesting their inherent flexibility (Figure S4). Meanwhile, the density maps provided sufficient detail for accurate modeling of HCAs’ ligands (Figure 1b). The acifran-HCA2-Gi and MK6892-HCA2-Gi complexes demonstrated conformations akin to the 3,5-DHBA-HCA1-Gi complex, with root-mean-square deviation (rmsd) values of 0.8 and 0.8 Å, respectively. Meanwhile, the acifran-HCA3-Gi complex yielded an rmsd value of 0.8 Å relative to the 3,5-DHBA-HCA1 complex (Figure S5a). It is evident that the ECL1–3 regions of HCA2 and HCA3 exhibit significant divergence from those of HCA1 (Figure S5a). This divergence is consistent with their distinct ligand recognition capacities.

The density maps robustly support accurate modeling of the ECL2 region across all receptors, revealing a conserved β-hairpin structure at the N-terminus of ECL2 that spans the ligand-binding pocket (Figure 1c). Within the extracellular region of HCA1, three disulfide bonds are established. Notably, the conserved disulfide linkage between C88^3.25^ and C165^45.50^, presented in both HCAs and Class A GPCRs, is instrumental in mediating the interaction between ECL2 and TM3 (Figures 1c, S5c, and S7). Additionally, the disulfide bridges formed by C7^N-ter^ with C252^ECL3^ and C6^N-ter^ with C157^ECL2^ establish connectivity with the N-terminus. Distinctively, the disulfide bond involving C6^N-ter^ and C157^ECL2^, which differentiates the orientation of the ECL region in HCA1 from that in HCA2 and HCA3, is replaced by C18^N-ter^ and C183^ECL2^ in the latter two receptors (Figure 1c). Upon individually disrupting these three pairs of disulfide bonds in HCA1, we observed that, aside from the conserved C88^3.25^ and C165^45.50^ bond whose disruption abolished ligand activation response, the disruption of the other two disulfide bonds had minimal impact (Figure 1d). This contrasts with the scenario in HCA2,^29,30,35,37^ where the disruption of any of the three disulfide bonds completely abolished activation, suggesting a greater flexibility at the N-terminus of HCA1.

**Figure 2:**
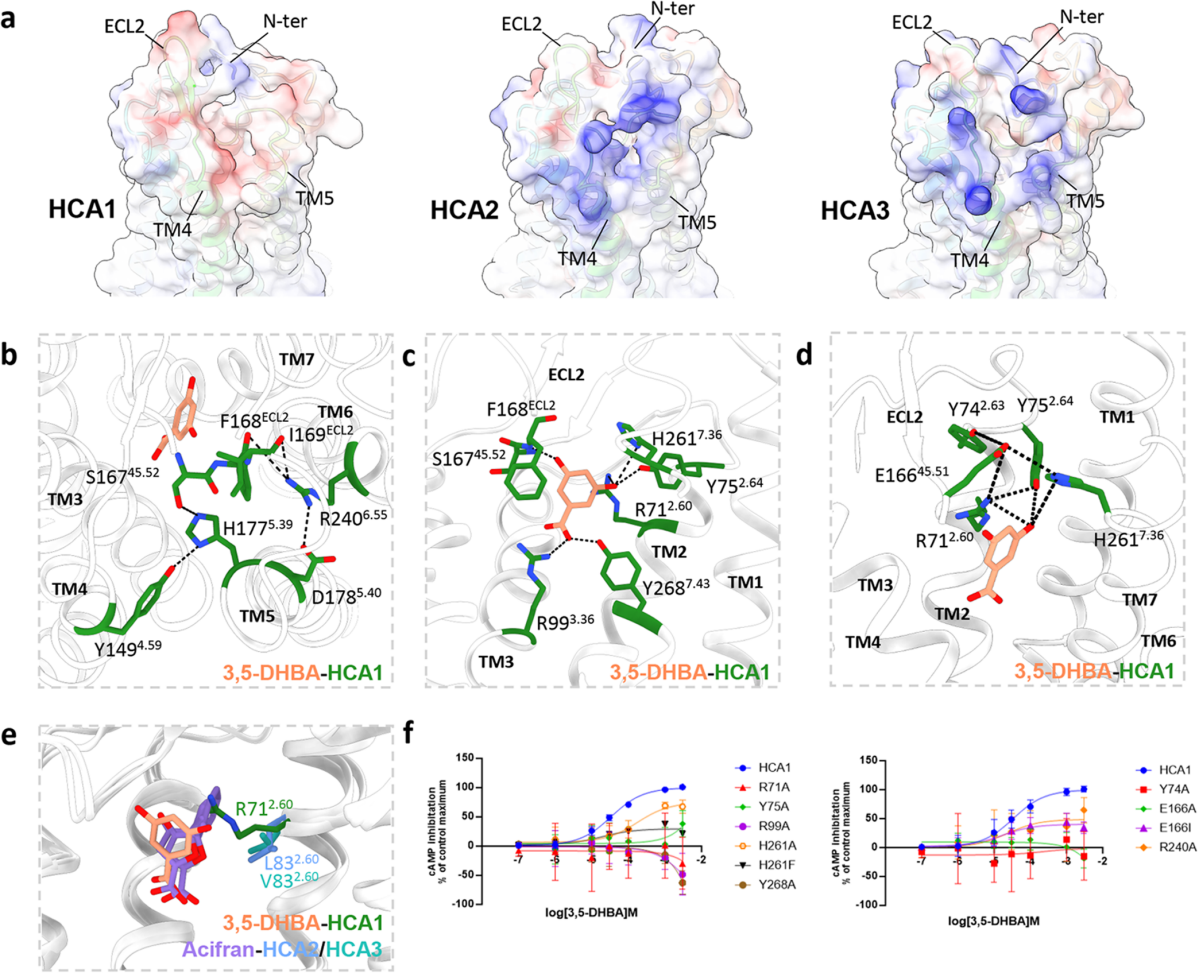
Orthosteric ligand-binding pocket of HCA1. (a) Sideview of HCAs with surface colored by electrostatic potential between TM4–5 and ECL2. Red is shown as negative potential, white as zero potential, and blue as positive. (b–d) Detail interactions around the orthosteric pocket in 3,5-DHBA-HCA1 (PDB: 9KT9). The black dotted line represents H-bonds and ionic interactions. Residues within 5 Å are labeled in these interactions and shown in stick representation. (e) Superimposition of ligand-bound HCAs. R71^2.60^ in HCA1 (PDB: 9KT9) narrows the space of the orthosteric pocket, compared with leucine and valine in HCA2 (PDB: 9KT8) and HCA3 (PDB: 9KT6), respectively. (f) HCA1 response curves induced by 3,5-DHBA. cAMP response was normalized to the wide type of HCA1. Data are shown as means ± SEM from three independent experiments, which were performed in triplicate.

In addition, we identified a negatively charged cavity within HCA1, characterized by a network of polar interactions involving ECL2, TM4, and TM5, in contrast to a positively charged cavity observed in HCA2/3 (Figure 2a,b). R240^6.55^ establishes polar interactions with the backbone of F168^ECL2^ and I169^ECL2^ in ECL2, thereby securing the position of ECL2 and stabilizing the ligand-binding pocket in HCA1 (Figure 2b). Following the mutation of R240^6.55^ to alanine, we found that the activation efficacy of 3,5-DHBA was reduced by around 50%, aligning with the attenuated efficacy of R251^6.55^A in HCA2^31^ (Figure 2f). Besides, Y149^4.59^ forms a connection with H177^5.39^ to bridge TM4 and TM5 in HCA1 and meanwhile, H177^5.39^ interacts with S167^45.52^ to connect TM5 and ECL2 in HCA1, mirroring the behavior of H189^5.39^ in HCA2/3 (Figures 2b and S5f). Given the role of H189^5.39^ as a dynamic gate for the entry/exit pathway in HCA2,^36^ we propose that the linkage between Y149^4.59^ and H177^5.39^ contributes to the stabilization of the closed state during HCA1 activation.

The conserved Cx^45.51^SF motif is prominently situated above the ligand-binding pocket within the ECL2 of HCAs, wherein the cysteine forms a conserved disulfide bond with TM3, and the phenylalanine inserts into the binding site (Figures 2c and S5b,e). A complex network of polar interactions involving E166^45.51^, H261^7.36^, Y74^2.63^, Y75^2.64^, and R71^2.60^ is formed in HCA1, whereas hydrophobic residues occupy the analogous positions of 45.51 in HCA2 and 7.36 in HCA2/3, delineating a more constricted cleft between TM2, TM7, and ECL2 in HCA1 (Figures 2d and S5d). Mutagenesis and cellular functional assays revealed that the substitution of these single critical residues with alanine in HCA1 significantly diminished the receptor’s responsiveness to 3,5-DHBA (Figure 2f). Specifically, the mutation of H261^7.36^ to phenylalanine, akin to the residues in HCA2/3, resulted in a marked decrease of efficacy compared with H261^7.36^A (Figure 2f), emphasizing the significance of the hydrophilic property of H261^7.36^ over its spatial occupancy. Conversely, in contrast to the inactivity following mutation to alanine, which breaks the polar interaction with R71^2.60^ and H261^7.36^, mutating E166^45.51^ to isoleucine (corresponding to 45.51 in HCA3) resulted in a halving of the activation efficacy compared with the wild type, suggesting that the spatial occupancy of the E166^45.51^ side chain is more critical than its polarity (Figure 2f). These observations suggest that the polar interactions and disulfide bonds underpin the stable conformation of ECL2 upon ligand activation and may play a crucial role in the specific recognition of HCAs.

### Ligand-Binding Site of HCA1

Our structure reveals that the 3,5-DHBA-HCA1 complex harbors a binding pocket analogous to that observed in HCA2/3, situated between TM2, TM3, and TM7 and sealed by ECL2 (Figure 2c). The side chain of F168^ECL2^ in HCA1 penetrates the ligand pocket, consistent with observations previously reported for HCA2/3 (Figure S5e). The ligand-binding pocket is surrounded by a set of hydrophobic residues, including Phe168^ECL2^, Leu264^7.39^, Leu92^3.29^, and Leu95^3.32^, conserved among HCAs (Figure S6c). The negatively charged carboxyl group of 3,5-DHBA delves into the pocket, establishing a robust salt bridge with R99^3.36^ and forming a hydrogen bond with Y268^7.43^. Additionally, the backbone of S167^45.52^ engages with the hydroxyl moiety of the agonist, a conserved interaction across the HCA family (Figures 2c and S5e).

3,5-DHBA and 3Cl-5OH-BA represented as the synthetic ligands of HCA1 perform specifical activity with an EC_50_ value of ∼150^23^ and ∼16 μM,^3,38^ while lactate as the endogenous agonist for HCA1 with a reported EC_50_ value range of 1–5 mM.^3,38,39^ Meanwhile, both 3,5-DHBA and lactate exhibit specific activity within HCA1^1,13,23,40^ (Figure S2d and Table S2). Moreover, we conducted docking simulations of the endogenous agonist lactate into HCA1 (Figure S6a,b) and compared it with the structure of the 3,5-DHBA-HCA1 complex. It was discovered that, in comparison to lactate, 3,5-DHBA is specifically stabilized within the ligand pocket through three additional hydrogen bonds formed between interactions with R71^2.60^, Y75^2.64^, H261^7.36^, and the 5′-hydroxyl group (Figure 2c). These interactions elucidate the enhanced potency of 3,5-DHBA (pEC_50_ = 3.7 ± 0.1) relative to lactate (pEC_50_ = 3.2 ± 0.3) (Table S2a,b). Remarkably, R71^2.60^ in the HCA1-Gi complex, distinct from Leu83^2.60^ in HCA2 and Val83^2.60^ in HCA3, is conserved among species.^41^ Upon superimposing the receptor structures, it is observed that the side chain of R71^2.60^ occupies a space typically reserved for agonists in HCA2 and HCA3, consequently leading to a reduced volume within the ligand-binding pocket of HCA1 (Figure 2e). Alanine substitutions of these critical residues lead to the complete abrogation of the ligand activity, highlighting their indispensable role in ligand recognition for HCA1 (Figure 2f). Furthermore, R99^3.36^ features as the crucial residue in the ligand binding of HCA1, similar to its role in HCA2. The HCA1 R99A mutation results in a complete loss of potency in response to both lactate and 3,5-DHBA (Figure 2f).^40^

Considering that 3-HBA can activate both HCA1 (EC_50_ = ∼184 μM) and HCA2 (EC_50_ = ∼215 μM),^3,22^ whereas 3,5-DHBA selectively activates only HCA1, we also undertook docking simulations of 3Cl-5OH-BA into HCA1 and 3-HBA into both HCA1 and HCA2, observing similar interactions between agonists and S^45.52^, R^3.36^, and Y^7.43^ (Figure S6b). The sole difference between 3-HBA and 3,5-DHBA is the presence of the 5′-hydroxyl group, providing additional interactions with R71^2.60^, Y75^2.64^, and H261^7.36^ (Figures 2c and S6a). R71^2.60^ minimizes the room for the ligand binding and meanwhile, the interaction network formed by Y74^2.63^, Y75^2.64^, E166^45.51^, and H261^7.36^ narrows the ligand pocket between TM2/7 and ECL2, making more extensive connections and highlighting the significant role of aforementioned residues in HCA1 for the recognition of selective agonists (Figure 2d,e). Our structure also explains that 3Cl-5OH-BA, a halogenated derivative of 3,5-DHBA, may exhibit a halogen bond with Y75^2.64^, while conserved interactions are formed with R99^3.36^, S167^45.52^, and Y268^7.43^, demonstrating enhanced ligand activation potency (Figure S6b). Therefore, these findings offer profound insights of specific ligand recognition by HCA1.

### Identification of Key Amino Acids for Selective Ligand Recognition in HCA2 and HCA3

**Figure 3:**
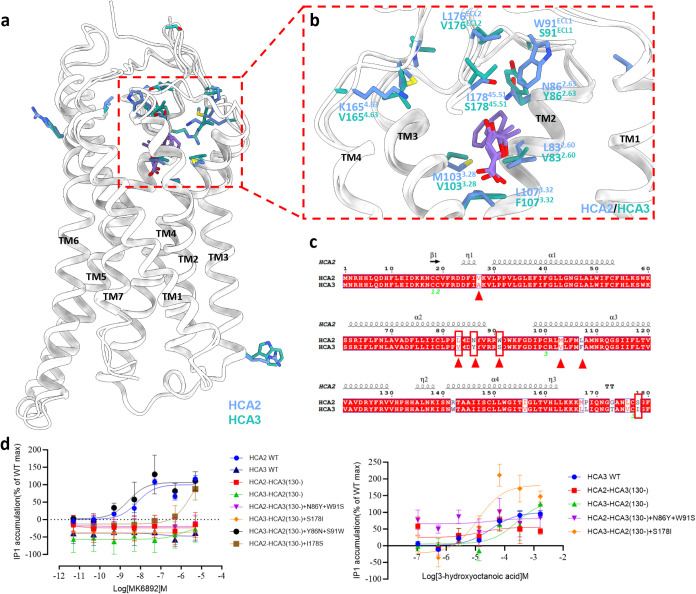
Chimeric receptor between HCA2 and HCA3. (a) Side view of acifran-bound HCA2 (PDB: 9KT8), aligned with acifran-HCA3 (PDB: 9KT6). Different residues are highlighted in the stick presentation. Most of the residues are around the ligand-binding pocket, facilitating specific ligand selectivity. (b) Structural comparison of residues around the ligand-binding pocket in HCA2/3. Acifran-HCA2 is in medium slate blue, and acifran-HCA3 is in light sea green. (c) Sequence alignment of residues in HCA2 and HCA3 in the first four transmembrane (TM) domains. The distinguished residues in the first three TM domains are highlighted by a red triangle, and the residues for single mutation are labeled by a red rectangle. (d) IP1 accumulation assay of chimeras of HCA2/HCA3, induced by MK6892 and 3-HO, respectively.

HCA2 and HCA3 differ by merely 16 amino acids, yet they recognize distinct ligands. The agonist 3-hydroxy-octanoic acid (3-HO) specifically activates HCA3, whereas HCA2 is selectively activated by MK6892. To investigate the differential mechanisms behind their ligand-selective recognition, a comprehensive structural comparison was undertaken. This analysis disclosed that, despite the minimal amino acid sequence divergence between HCA2 and HCA3, significant disparities were observed in the residues within the hydrophobic ligand-binding pocket of the TM3, ECL1, and ECL2 regions (Figures 3a–c and S7). Consequently, we opted to swap the amino acid segments 1–129 between HCA2 and HCA3, creating chimeras HCA2(1–129)/HCA3(130-) and HCA3(1–129)/HCA2(130-). Neither chimera was activated by the selective agonist MK6892. Activation was achieved only with the introduction of double mutations Y86^2.63^N and S91^ECL1^W in the chimera HCA3(1–129)/HCA2(130-), highlighting the crucial role of positions N86^2.63^ and W91^ECL1^ in HCA2 for selective ligand binding (Figure 3d). Conversely, even though the ligand 3-HO failed to activate chimera HCA2(1–129)/HCA3(130-), it was capable of activating HCA3(1–129)/HCA2(130-), with the mutation S178^45.51^I displaying the highest potency (Figure 3d). Moreover, in the chimera HCA2(1–129)/HCA3(130-), mutation of N86^2.63^ and W91^ECL1^ to the corresponding residues Tyrosine and Serine in HCA3 did not facilitate activation by 3-HO (Figure 3d). These results suggest that the first three TMs along with I178^45.51^ in HCA3 play a pivotal role in the selective recognition of its ligands.

Consequently, although the HCA family exhibits conserved key residues within the orthosteric binding pocket, R71^2.60^, Y74^2.63^, Y75^2.64^, E166^45.51^, and H261^7.36^ play a crucial role in establishing a more compact and stable ligand-binding pocket specifically in HCA1 (Figure 2c,d). In HCA2, the essential residues N86^2.63^ and W91^ECL1^ are critical for selective ligand recognition. In contrast, V83^2.60^, Y86^2.63^, S91^ECL1^, V103^3.28^, F107^3.32^ (residues situated within the binding pocket of the first three transmembrane regions that vary from HCA2), and I178^45.51^ are posited to serve analogous roles in HCA3 (Figure 3c).

### Possible Binding Mode of AZ2 and Compound 2 in HCA1

**Figure 4:**
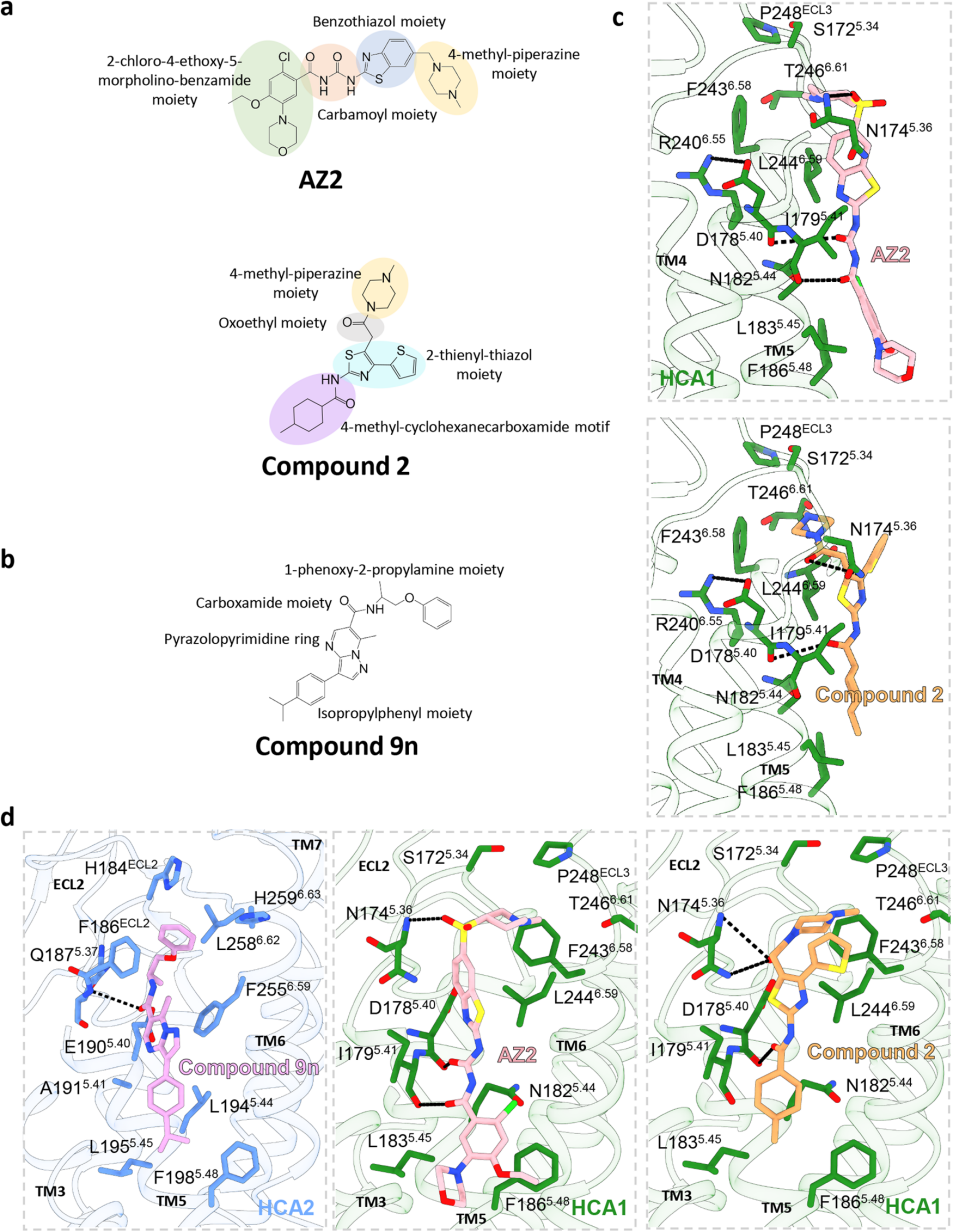
Molecular docking of AZ2 and compound 2 in HCA1. (a) Detailed chemical structure of AZ2 and compound 2. Different circles represent distinct groups of small molecules. (b) Chemical structure of compound 9n, with the groups labeled around. (c, d) The side view shows the structure of molecular docking of the ligand-binding pocket of AZ2 and compound 2, compared with the allosteric pocket of compound 9n-HCA2 (PDB: 8JHY).

AZ2 and compound 2 have previously been identified as agonists for HCA1, exhibiting EC_50_ values of ∼70–180 and ∼50 nM,^14,24^ respectively. However, the orthosteric ligand pocket of HCA1 between TM1, TM2, TM3, TM7, and ECL2 seems insufficiently spacious to accommodate AZ2 and compound 2. Besides, the absence of a carboxyl group in AZ2 and compound 2, in contrast to HCA1 ligands, suggests that interaction with R99^3.36^ in HCA1 may be difficult to form (Figure S2a). Since their chemical structures both contain a methyl-piperazine moiety as the headgroup and several amphipathic groups in the middle, we suppose that they may perform with a resemble binding mode during the activation of HCA1 (Figure 4a). Concurrently, the structure of compound 9n presents a group distribution similar to those of AZ2 and compound 2 (Figure 2a,b). Based on studies regarding the allosteric modulation of HCA2 by compound 9n,^29,33^ we hypothesize that AZ2 and compound 2 may act as biased agonists for HCA1. Subsequently, we docked AZ2 and compound 2 into the analogous positions in HCA1 that correspond to those of HCA2 (Figure 4d). Both HCA1 and HCA2 feature a salt bridge between D/E^5.40^ and R^6.55^, linking TM5 and TM6 (Figure 4c).^33^ According to the docking structure, the methyl-piperazine moiety of AZ2 and compound 2 penetrates the hydrophilic cavity formed by TM5 and TM6, similar to the binding mode observed for compound 9n in HCA2. Compound 9n forms a single hydrogen bond between its carboxamide moiety and Q187^5.37^, while its isopropylphenyl moiety is embedded in the hydrophobic pocket of TM5 (Figure 4d). Similarly, AZ2 and compound 2 also form stable interactions with N174^5.36^ and D178^5.40^. In addition to their insertion into the hydrophobic pocket, these interactions suggest a high possibility of allosteric agonist regulation within HCA1 (Figure 4d).

### Active Conformation of HCAs

**Figure 5:**
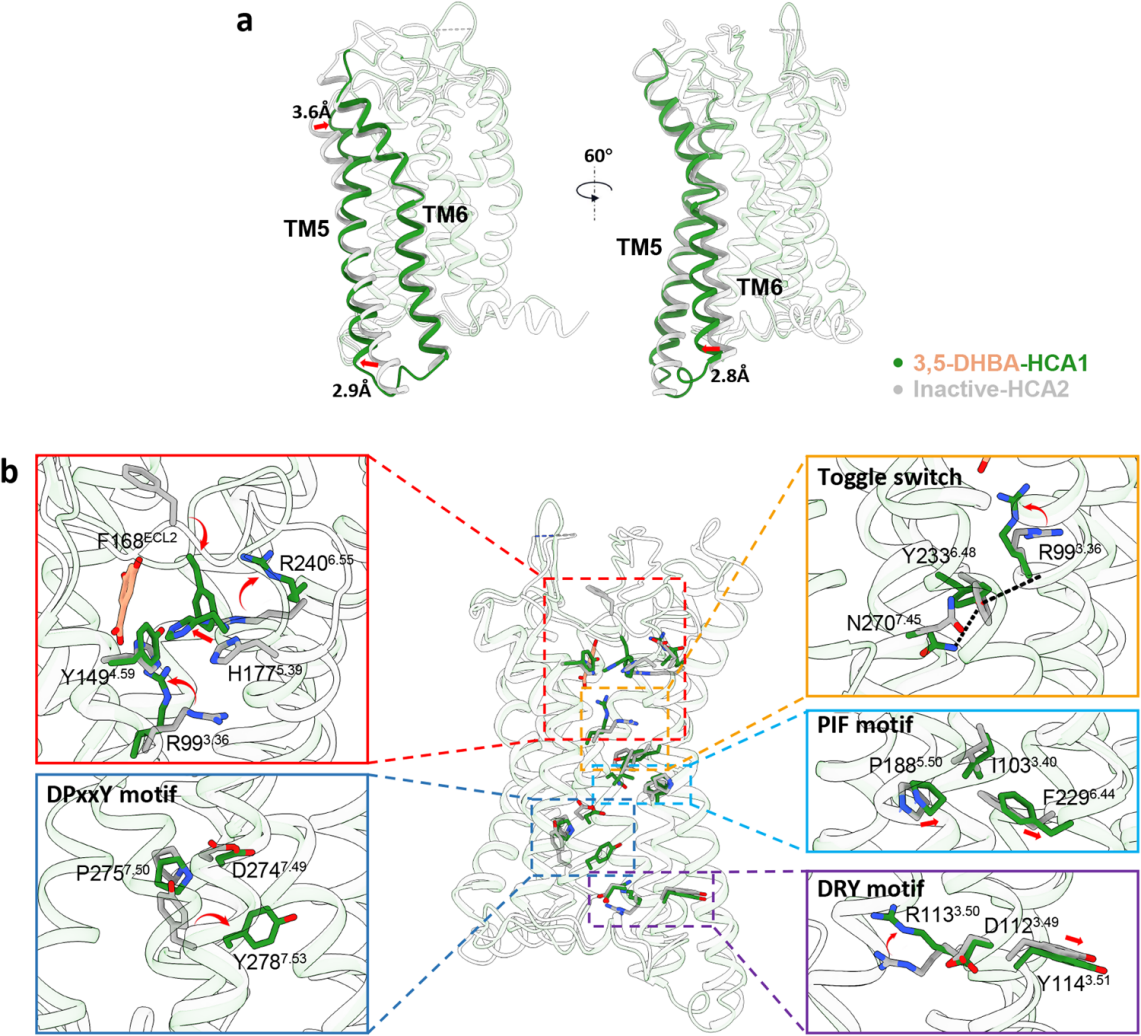
Activation of HCA1. (a) Superimposition of the active conformation of HCA1 (forest green, PDB: 9KT9) with the inactive conformation of HCA2 (light gray, PDB: 7ZLY). (b) Detailed side view of the rearrangement of the conserved residue R99^3.36^ in HCAs, together with conserved motifs (toggle switch, PIF motif, DPxxY motif, and DRY motif), compared with the inactive state of HCA2. Toggle switch is labeled in yellow rectangle, PIF motif in blue, DRY motif in purple, and DPxxY motif in dark blue. Distinct rotation and movements are presented by red arrows.

To elucidate the activation mechanisms of HCA1 further, we performed pairwise comparisons of our structures versus the inactive structure of HCA2 (Figure 5a). This comparison unveiled conserved conformational shifts, encompassing the inward movement of the extracellular segment of TM5 and the outward displacement of TM5 and TM6 within the cytoplasmic domain. Compared with the apo state of HCA2, the extracellular portion of TM5 in agonist-bound HCA1 exhibited a displacement of 3.6 Å, which is less than the 5.5 Å observed in HCA2 and 6.1 Å in HCA3. Additionally, the intracellular region of TM5 in HCA1 moved outward by 2.9 Å, also less than the 4.2 Å in HCA2 and 4 Å in HCA3. Meanwhile, TM6 in the intracellular region of all of these receptors exhibited an outward movement of about 2.3–2.8 Å (Figures 5a and S8a). Agonist activation prompts a rotational adjustment of approximately 90° in R99^3.36^ to engage with agonist, while R240^6.55^ similarly rotates, facilitating a reorientation of the F168^ELC2^, thereby occluding the binding site. Concurrently, the relocation of F168^ECL2^ toward the ligand-binding pocket necessitates the outward repositioning of TM6 to mitigate steric interference with the Gi protein (Figure 5b). Meanwhile, the conformational rearrangement during the activation of HCA3 is also consistent with that of HCA2, involving the rotation of R111^3.36^, R251^6.55^, and F180^ECL2^, suggesting the conservation of the activation mechanism across the HCA family (Figure S8b,c).

In alignment with HCA2 and HCA3, activation triggers a cascade of conformational alterations in ECL2 and canonical motifs (PIF, DPxxY, and DRY motif) within HCA1 (Figures 5b and S8b,c). In particular, the toggle switch motif in HCA1 is Tyr233^6.48^ instead of Phe in HCA2/3 and Trp in the majority of class A GPCRs (Figure 5b). As anticipated, Tyr233^6.48^ in HCA1 undergoes a 90-degree rotation. This results in the formation of hydrogen bonds with the side chain of N270^7.45^ and the backbone of R99^3.36^, specifically involving the side chain of Tyr233^6.48^ in HCA1, which facilitates signal transduction and stabilizes the receptor in its active conformation.

### Interactions between HCA and Gi Protein

**Figure 6:**
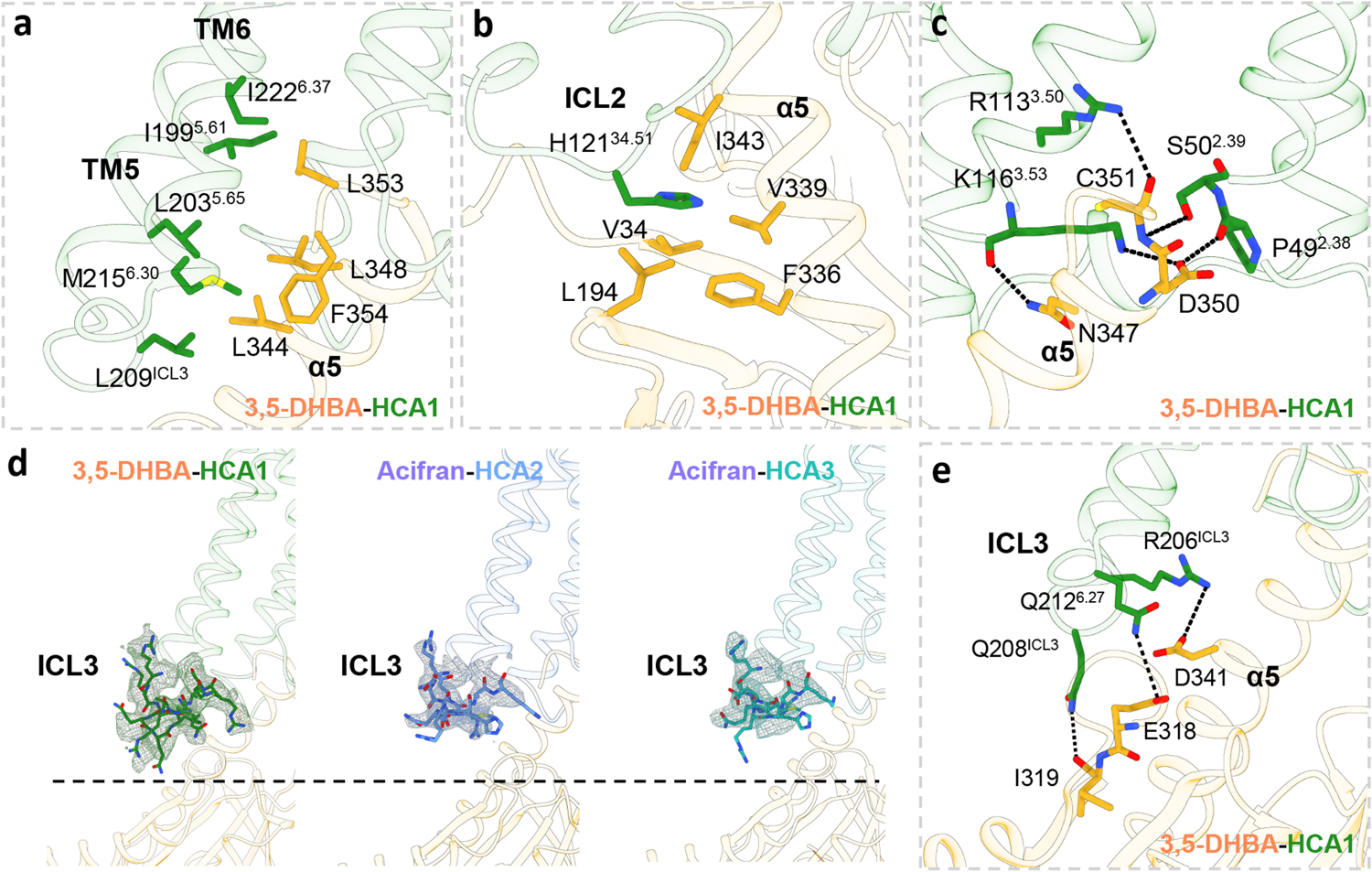
Interactions between HCA1 and Gi. (a) The hydrophobic interactions formed between TM5–6 of HCA1 and α5 of Gαi (PDB: 9KT9). (b) The hydrophobic pocket around H121^34.51^, which is conserved in HCAs. (c) An interlock network formed by H-bonds and polar interactions in ICL1, TM3, and α5 of Gαi. (d) ICL3 comparison between HCA1, HCA2, and HCA3. (e) Detail connections formed by ICL3 in HCA1 and α5 of Gαi. The black dotted line represents H-bonds and ionic interactions. Residues within 5 Å are labeled in these interactions and shown in stick representation.

The structure of the 3,5-DHBA-HCA1-Gi complex exhibits a conserved global architecture, with the principal interaction interface constituted of TM3, TM5, and TM6, alongside ICL2 and ICL3 of HCA1 and the α5 helix and αN helix of the Gαi protein (Figure 6a–c).

A critical hydrophobic interface around the α5 helix domain of the Gαi protein is composed of a series of hydrophobic residues (I199^5.61^, L203^5.65^, L209^ICL3^, M215^6.30^, and I222^6.37^ in HCA1 and L344, L348, L353, and F354 in Gαi), consistent with HCA2/3 (Figures 6a and S9a,d). The conserved residues H121^34.51^ in HCA1 and H133^34.51^ in HCA2/3 are embedded into the hydrophobic pocket of the Gαi protein (Figures 6b and S9b,e). Besides, a set of conserved interactions at the interface between HCAs and Gαi are observed (Figures 6c and S9c,f): (i) the backbone of K116^3.53^ in HCA1 and R128^3.53^ in HCA2/3 interacts with the side chain of N347, while the side chain of 3.53 also interacts with the side chain of D350. (ii) The side chain of serine in 2.39 in HCA1–3 (S50 in HCA1 and S62 in HCA2/3) forms a hydrogen bond with the backbone of C351. The interaction between the side chain of R128^3.53^ and the backbone of N347 is pronounced in HCA2/3, absent in HCA1 (Figures 6c and S9c,f). Moreover, R113^3.50^ interacts with the main chain of C351 in HCA1, diverging from the interaction between R63^2.40^ and the main chain of C351 in HCA2. Besides, P49^2.38^ interacts withD350 in HCA1, while S61^2.38^ in HCA2 interacts with R128^3.53^ (Figures 6c). Conversely, ICL3 in HCA1 penetrates more deeply into the cytoplasm compared with HCA2 and HCA3 (Figure 6d), facilitating an expanded polar interaction interface with the Gαi protein, including engagements between R206^ICL3^, Q208^ICL3^, and Q212^6.27^ in HCA1 and D341, and I319 and E318 in Gαi, respectively (Figure 6e). In HCA2 and HCA3, R218^5.68^ interacts with D337 and D341 on the α5 helix of the Gαi protein, while R222^ICL3^ in HCA3 additionally formed a salt bridge with E318 in the Gαi protein (Figure S9g,h).

Furthermore, a notable divergence exists between the C-terminus of HCA1 and that of HCA2/3: In HCA1, residue T51^2.40^ replaces R63^2.40^ found in HCA2/3, precluding the formation of a hydrogen bond between R63^2.40^ and C351 in the Gαi protein. Alternatively, with the spatial steric hindrance of the R63^2.40^ side chain absent, residues F285^8.50^ and Y279^7.54^ in HCA1 undergo a rotation of approximately 90 degrees relative to their analogous positions in HCA2 (Figure S9i).

## Discussion

Within the HCA family, HCA1 is the phylogenetically eldest receptor identified in fish, while HCA2 emerged in mammals, and HCA3 was exclusively found in higher primates. Moreover, 3,5-DHBA specifically activates HCA1, whereas MK6892 and β-hydroxy-octanoic acid are selectively targeted toward HCA2 and HCA3, respectively. Given the adverse effect of cutaneous flushing associated with treatments targeting HCA2, activating HCA1 for antilipolysis without side effects has been explored. Heretofore, the structures of apo-state HCA2 and HCA2 bound with diverse ligands, such as niacin, acipimox, and GSK256073, as well as allosteric ligand compound 9n have been investigated for the ligand recognition and activation mechanism of HCA2.^28−37^ Meanwhile, acifran bound with HCA3 has also been explored for the investigation of ligand selectivity between HCA2 and HCA3.^31^ However, there is still no information for the structure of HCA1 yet, resulting the shared and unique features of the selectivity mechanisms for agonist recognition across all three receptors remained elusive. To advance our comprehension of HCA’s functional mechanisms, we present four structures: 3,5-DHBA-HCA1, acifran-HCA2, MK6892-HCA2, and acifran-HCA3 complexes with the Gi protein.

Through the integration of structural studies with mutational analyses, we aim to clarify the structure–function relationships in agonist-bound HCAs-Gi complexes. Three disulfide bonds, along with a series of polar interactions, secure the position of ECL2, which caps the ligand-binding pocket and stabilizes the extracellular domain. Distinctly, HCA1 forms a negatively charged cavity, in contrast to the positively charged cavity in HCA2/3. Intriguingly, E166^45.51^ in HCA1 links ECL2 with R71^2.60^, Y74^2.63^ in TM2, and H261^7.36^ in TM7, thus creating a narrow entrance above the ligand-binding pocket. Additionally, HCAs’ ligands, characterized by a hydroxyl group, engage with the conserved R^3.36^, Y^7.43^, and the backbone of S^45.52^ across all HCAs. The additional interaction of the 5′-hydroxyl group in 3,5-DHBA with R71^2.60^, Y75^2.64^, and H261^7.36^ in HCA1 highlights its pronounced selectivity for HCA1. Particularly, Arg71^2.60^ in HCA1, unlike Leu83^2.60^ in HCA2 and Val83^2.60^ in HCA3, narrows the ligand pocket, favoring smaller, more rigid agonists. Since the lack of molecular structure information on HCA1, there is no investigation focused on the critical residues during the specific ligand binding. Instead, only the residues including R71^2.60^ and R99^3.36^ have been identified to function in the activation of HCA1.^41^ According to our structure of 3,5-DHBA-HCA1, we suppose that the stable interaction network formed by R71^2.60^, Y74^2.63^, Y75^2.64^, E166^45.51^, and H261^7.36^ plays a crucial role in specific ligand recognition of HCA1. Previous research provide insights into several residues (L/V83^2.60^, N/Y86^2.63^, W/S91^ECL1^, M/V103^3.28^, L/F107^3.32^, and S/I178^45.51^) as key residues during the different specific ligand binding in HCA2/3.^34,42,43^ However, our chimeric HCA2/HCA3 constructs highlight N86^2.63^ and W91^ECL1^ as key residues for HCA2 specificity (MK6892), and the first three transmembrane domains along with I178^45.51^ as critical for HCA3 specificity (3-HO).

In addition, according to the molecular docking, we hypothesize the possible allosteric binding mode of AZ2 and compound 2 during the activation of HCA1. Besides, we investigate the conformational change in the receptor including the similar movement of canonical motifs, resulting in the classic change in TM5 and TM6, facilitating Gi binding. Our analysis of Gi coupling reveals that the deeper insertion in HCA1 fosters an extensive interaction network with Gi, and T51^2.40^ in HCA1 rotates approximately 90 degrees, leading to the disruption of a hydrogen bond observed in HCA2/HCA3.

In summary, our findings elucidate the molecular mechanisms underlying ligand recognition and G-protein activation in HCA1, identify key amino acids that influence the specificity of ligand recognition in HCA2 and HCA3, and provide detailed structural templates for the design and refinement of specific ligands targeting the HCAs receptor family.

## Methods

### Construct

The human HCA1/HCA2/HCA3 is cloned into the pFastBac plasmid with a hemagglutinin (HA) signaling peptide followed by a FLAG tag (DYKDDDD) at the N-terminus, and the bRIL fusion protein was added to the N-terminal of three GPCRs. In HCA1, a single mutant D274^7.49^N was used to improve the protein yield. Single-chain antibody scFv16 was cloned into the pFastBac vector containing a GP67 signal sequence at the N-terminus and a TEV cleavage site at the C-terminus before an 8 × His tag. For the IP1 accumulation assay, HCA1/HCA2/HCA3 is cloned into the pTT5 plasmid.

### Expression and Purification of HCA1/HCA2/HCA3-Gαi Fusion Protein

For protein expression, recombinant baculovirus of human HCA1/HCA2/HCA3, Gαs, His6-tagged Gβ1, and Gγ2 were coexpressed by infecting in *S. frugiperda* (Invitrogen) cells. The HighFive (Invitrogen) cells were infected at 2–3 × 10^6^ cell/mL density and cultured at 28 °C, 130 rpm. After 48 h, cells were collected by centrifugation and stored at −80 °C. For protein purification, cells were resuspended in binding buffer (20 mM HEPES, pH 7.5, 50 mM NaCl, 2 mM MgCl_2_) supplemented with protease inhibitor cocktail tablets (Roche), 25 mU mL^–1^ Apyrase, 10 mg mL^–1^ scFv16, and 1 mM 3,5-DHBA/20 μM Acifran/20 μM MK6892. The mixture was incubated for 1 h at room temperature and centrifugated at 38000 rpm for 30 min. Then membranes were solubilized in 25 mM HEPES, pH 7.5, 150 mM NaCl, 0.5% (w/v) lauryl maltose neopentyl glycol (LMNG, Anatrace), 0.025% cholesterol hemisuccinate (CHS, Anatrace), 2 mM MgCl_2_, 25 mU mL^–1^ Apyrase, and 1 mM 3,5-DHBA/20 μM Acifran/20 μM MK6892 at 4 °C for 2 h. After centrifugation, the supernatant was incubated with Strep-Tactin XT (IBA) resin overnight at 4 °C. The resin was washed with a buffer containing 25 mM HEPES, pH 7.5, 150 mM NaCl, 0.01% LMNG, 0.0005% CHS, 2 mM MgCl_2_, and 1 mM 3,5-DHBA/20 μM Acifran/20 μM MK6892 for about 20 column volumes. Then it was eluted with 50 mM biotin, 150 mM Tris-HCl, pH 8.0, 150 mM NaCl, 0.01% (w/v) LMNG, 0.0005% CHS, 2 mM MgCl_2_, and 1 mM 3,5-DHBA/20 μM Acifran/20 μM MK6892. The complex protein was concentrated and subjected to a Superdex 200 Increase 10/300 column (GE Healthcare) with 20 mM HEPES, pH 7.5, 100 mM NaCl, 0.01% (w/v) LMNG, 0.0005% CHS, 2 mM MgCl_2_, and 1 mM 3,5-DHBA/20 μM Acifran/20 μM MK6892.

### Expression and Purification of scFv16

The scFv16 baculovirus using the Bac-to-Bac system infected the Hi5 cells without serum at a density of 3 × 10^6^ cells/mL. After 48 h of incubation at 28 °C, cells were harvested by centrifugation at 4000 rpm for 30 min. The supernatant was combined with 5 mM CaCl_2_, 1 mM NiCl_2_, and 15 mM Tris, pH 8.0 for 1 h, then mixed with Ni Superflow resin (Smart-Lifesciences) and incubated for 2 h at 4 °C. The collected resin was washed with buffer containing 20 mM HEPES, pH 7.5, 500 mM NaCl, and 30 mM imidazole for 20 column volumes, and further washed with buffer containing 20 mM HEPES, pH 7.5, 100 mM NaCl, and 30 mM imidazole for 20 column volumes. Next, the 8 × His tag was cleaved overnight by 20 U/mg HRV-3C protease, and then scFv16 was eluted with buffer containing 20 mM HEPES, pH 7.5, and 100 mM NaCl. The sample was concentrated and loaded into a size-exclusion column (Superdex 75 Increase 10/300 GL, Cytiva) for purification. Peak fractions were concentrated to 10 mg/mL with a centrifugal filter device (cobetter 10-kDa MW cutoff) and stored at −80 °C until use.

### Cryo-EM Grid Preparation and Data Collection

For grid preparation, holey carbon grids (Quantifoil R1.2/1.3, Au 300mesh) were glow-discharged at 15 mA for 25 s. Three μL of purified ligand-bound HCAs in a complex of Gi proteins was applied to grids with a wait time of 5 s and a blot time of 3 s using Vitrobot Mark IV (FEI). Grids were then plunge-frozen into liquid ethane and transferred to a 300 keV Titan Krios G3i electron microscope (FEI) equipped with a Gatan K3 summit direct electron detector and Quantum energy filter for data collection. Cryo-EM images were recorded in super-resolution mode at a magnification of 105,000× and a defocus range of −1.0 to −1.5 μm, corresponding to a pixel size of 0.4255 Å. Micrographs were dose-fractioned with 40 frames with an accumulated dose of 54 e-/Å^2^.

### Cryo-EM Data Processing

All movies were imported into Relion 3.1.0 and performed with MotionCor for beam-induced motion correction with a binning of 2 and Gctf for contrast transfer function estimation.^44−46^ For the data set of 3,5-DHBA-HCA1-Gi complex, a subset of 2,605,173 particles was extracted from 4,126 movies and subjected to two-dimensional (2D) classification and two rounds of three-dimensional (3D) classification. 918,047 particles were selected for further 3D refinement, followed by postprocess, polish, and Ctf refinement, resulting in a final resolution of 2.70 Å. Signal subtraction was performed to obtain better resolution for the transmembrane domain of HCAs. For the acifran- and MK6892-HCA2-Gi complexes and the acifran-HCA3-Gi complex, 5732, 6900, and 3315 micrographs were collected for data processing, respectively. A total of 2,886,231, 5,044,785, and 3,145,848 particles were autopicked and the best classes were selected after 2D classification and 3D classification, which generated maps with resolutions at 2.80, 2.73, and 3.01 Å, respectively.

### Model Building and Refinement

The initial template of HCA1 was generated using AlphaFold2^47^ and coordinates as well as geometry restrain of ligands were generated using elbow, Phenix.^48^ Initial templates and ligand models were rigid-fitted into the maps using Chimera,^49^ followed by manual refinement in Coot.^50^ After iteration of refinement using Coot and Phenix, the final models were validated using Phenix. All of the figures were prepared with Chimera and ChimeraX.^51^

### Molecular Docking

First, the selection of the HCA1 docking model is crucial. The structure selected for orthosteric site docking is the HCA1 receptor binding 3,5-dihydroxybenzoic acid structure that we have analyzed using cryo-EM of 3,5-DHBA-HCA1 complex, the allosteric site docking selected the structure of HCA2 receptor binding 9n (PBD ID: 8J6Q),^29^ and used Swiss model^52^ to model its homology, obtaining a more reasonable docking model.

Autodock vine software^53^ was used to dock HCA1 receptors and a series of small molecules. First, the small molecules and proteins were preprocessed separately. The preprocessing for small molecules includes three-dimensional conformation generation, isomer generation, etc. The preprocessing for proteins includes hydrogenation, energy optimization, etc. After that, the protein pocket was set. The orthosteric site and allosteric site, respectively, selected 10 * 10 * 10 Å^3^ and 15 * 15 * 15 Å^3^ cubic boxes as docking pockets, the docking module was used to dock receptor and ligand, and the final docking result was selected through docking scoring and binding mode evaluation.

### IP1 Accumulation Assay

The HCA1/HCA2/HCA3 plasmid was transfected into the HEK293 cells (Invitrogen) and cultured for 2 days. The HEK293 cells expressing HCA1/HCA2/HCA3 were washed by PBS and resuspended by the stimulation buffer from a Cisbio Bioassays’ IP-One Gq kit. Then added 14000 cells to a 384-well plate in 7 μL buffer and added different concentrations of 3-hydroxycaprylic acid (3.3 mM to 0.213 μM) or MK6892 (10^–5^–10^–11^ M) for 7 μL. After incubation at 37 °C for 90 min, 3μl Tb Cryptate Antibody and 3μl IP1 d2 Reagent were added to 384-well plates per well. After incubation at room temperature for 1 h, the 384-well plate was read using a microplate reader (TECAN) with excitation at 320 nm and emission at 620 and 665 nm. The data was analyzed by GraphPad Prism 8.4.0 (GraphPad Software).

### Reporter Assay

Human embryonic kidney 293 (HEK293) cells were maintained in Dulbecco’s modified Eagle’s medium (DMEM) containing 10% fetal bovine serum (FBS) and antibiotics (100 U/mL penicillin and 100 μg/mL streptomycin) at 37 °C, 5% CO_2_. Cells (1 × 10^6^ per well) were seeded on 6-well plates for 15 h and transiently transfected with a transfection cocktail containing 2 μg of plasmid DNA (1 μg of the target plasmid and 1 μg of pCRE-luc) and 6 μL of X-tremeGENE HP DNA transfection reagents dissolved in 200 μL of phenol red-free OPTI MEM. After 24 h incubation, the medium containing the transfection cocktail was replaced with a serum-supplemented normal culture medium, and cells were plated at a density of approximately 40000 cells/well in white opaque 96-well microplates for an additional 24 h. After 24 h, fresh medium with varied concentrations of ligands was added to the wells, and cells were incubated for 30 min at 37 °C, 5% CO_2_. Then, forsklin with a final concentration of 5 μM was added to each well for 6 h. Then, all of the medium was removed and 40 μL of Bright-Glo Luciferase Assay System reagent (Promega, U.K.) was added to each well. Luminance was measured in a luminometer (TECAN Spark, CH) after 10 min incubation.

## Supporting Information Available

Supplementary figures and tables, comparison of amine receptor structures, molecular docking results, and structure data processing model building and refinement (PDF)
Docking model of AZ2-HCA1 (PDB)
Docking model of compound 2-HCA1 (PDB)
Docking model of 3-HBA-HCA1 (PDB)
Docking model of lactate-HCA1 (PDB)
Docking model of 3-HBA-HCA2 (PDB)

## Supplementary Material

jm4c02567_si_001.pdf

jm4c02567_si_002.pdb

jm4c02567_si_003.pdb

jm4c02567_si_004.pdb

jm4c02567_si_005.pdb

jm4c02567_si_006.pdb

## Abbreviations Used

HCAs: hydroxy-carboxylic acid receptors
GPCR: G-protein-coupled receptor
cAMP: cyclic adenosine monophosphate
NLRP3: NOD-, LRR-, and pyrin domain-containing protein 3
3-HBA: 3-hydroxybutyric acid
3,5-DHBA: 3,5-dihydroxybenzoic acid
3Cl-5OH-BA: 3-chloro-5-hydroxy-benzoic acid
AZ2: 2-chloro-4-ethoxy-*N*-[[6-[(4-methylpiperazin-1-yl)methyl]-1,3-benzothiazol-2-yl]carbamoyl]-5-morpholino-benzamide
Compound 2: 4-methyl-*N*-(5-(2-(4-methylpiperazin1-yl)-2-oxoethyl)-4-(2-thienyl)-1,3-thiazol-2-yl)cyclohexanecarboxamide
7w-2: peptide with a sequence of SFHFGRL
N2L: *N*-(2-(5-(1,2-dithiolan-3-yl)pentanamido)ethyl)nicotinamide
3-HO: 3-hydroxy-octanoic acid
cryo-EM: cryo-electron microscopy
ECL: extracellular loop
ICL: intracellular loop
TM: transmembrane
BRIL: apocytochrome b562 fusion protein
