# Correction to “Insights into the Activation Mechanism of HCA1, HCA2, and HCA3”

**DOI:** 10.1021/acs.jmedchem.5c03287

**Published:** 2025-11-26

**Authors:** Jiening Wang, Yuxia Qian, Zhen Han, Yize Wang, Yanru Liu, Jie Li, Qingmiao Duanmu, Sheng Ye, Anna Qiao, Shan Wu

In the “**Expression and and Purification of HCA1/HCA2/HCA3-Gαi Fusion Protein**” section of the Methods, line 1 and 2 is here corrected to change “For protein expression, recombinant baculovirus of human HCA1/HCA2/HCA3, **Gαs**, His6-tagged Gβ1, and Gγ2 were coexpressed by infecting in *S. frugiperda* (Invitrogen) cells.” to “For protein expression, recombinant baculovirus of human HCA1/HCA2/HCA3, **Gαi**, His6-tagged Gβ1, and Gγ2 were coexpressed by infecting in *S. frugiperda* (Invitrogen) cells.”.

In the **Acknowledgments**, line 2 and 3 is here corrected to change “This work was supported in part by **the Ministry of Science and Technology** (2020YFA0908400 to S.W., 2023YFA0916300 to A.Q., and 2020YFA0908500 to S.Y.)” to “This work was supported in part by **the National Key Research and Development Program of China** (2020YFA0908400 to S.W., 2023YFA0916300 to A.Q., and 2020YFA0908500 to S.Y.)”.

