# Supplementary material for: Insights into the Activation Mechanism of HCA1, HCA2, and HCA3
Source: J Med Chem. 2025 Feb 12;68(4):4527–39. doi: 10.1021/acs.jmedchem.4c02567 (PMC11873900; doi:10.1021/acs.jmedchem.4c02567)
Supplement: Supplementary file 1 — jm4c02567_si_001.pdf [file jm4c02567_si_001.pdf]

Supporting Information

Insights into the activation mechanism of HCA1，HCA2 and HCA3

Jiening Wang[1#], Yuxia Qian[2#], Zhen Han[2#], Yize Wang[2], Yanru Liu[2], Jie Li[1], Qingmiao Duanmu[1], Sheng Ye[2*], Anna Qiao[2*], Shan Wu[1*]

1 State Key Laboratory of Biocatalysis and Enzyme Engineering, Hubei Collaborative Innovation Center for Green Transformation of Bio-Resources, Hubei Key Laboratory of Industrial Biotechnology, School of Life Sciences, Hubei University, Wuhan, Hubei 430062, China;

2 Tianjin Key Laboratory of Function and Application of Biological Macromolecular Structures, School of Life Sciences, Tianjin University, 92 Weijin Road, Nankai District, Tianjin 300072, China;

Corresponding Authors Information:

Shan Wu, School of Life Sciences, Hubei University, 368 Youyi Avenue, Wuchang District, Wuhan, Hubei 430062, China; E-mail: wushan91@hubu.edu.cn

Anna Qiao, School of Life Sciences, Tianjin University, 92 Weijin Road, Nankai District, Tianjin 300072, China; E-mail: anna.qiao@tju.edu.cn

Sheng Ye, School of Life Sciences, Tianjin University, 92 Weijin Road, Nankai District, Tianjin 300072, China; E-Mail: sye@tju.edu.cn

Authors Information:

Jiening Wang–State Key Laboratory of Biocatalysis and Enzyme Engineering, Hubei Collaborative Innovation Center for Green Transformation of Bio-Resources, Hubei Key Laboratory of Industrial Biotechnology, School of Life Sciences, Hubei University, Wuhan, Hubei 430062, China;

Yuxia Qian –Tianjin Key Laboratory of Function and Application of Biological Macromolecular Structures, School of Life Sciences, Tianjin University, 92 Weijin Road, Nankai District, Tianjin 300072, China;

Zhen Han –Tianjin Key Laboratory of Function and Application of Biological Macromolecular Structures, School of Life Sciences, Tianjin University, 92 Weijin Road, Nankai District, Tianjin 300072, China;

# These authors contribute equally to this work

Figure S1 | The purification of 3,5-DHBA-HCA1, MK6892-HCA2, acifran-HCA2 and acifran-HCA3. 3
Figure S2 | Chemical structure of ligands of HCAs. 4
Figure S3 | The cryo-EM data processing of 3,5-DHBA-HCA1, MK6892-HCA2, acifran-HCA2 and acifran-HCA3. 5
Figure S4 | Representative cryo-EM density of HCAs. 6
Figure S5 | The model of HCAs bound with agonists. 7
Figure S6 | Molecule dock of lactate-HCA1 and 3-HBA with HCA2. 8
Figure S7 | Sequence alignment of human HCA receptors. 9
Figure S8 | Activation of HCAs. 10
Figure S9 | The interactions between HCA2/HCA3 and Gi. 11
Figure S10 | Measurement of the cell surface expression level of mutant HCA1 constructs. 12
Table S1 | Agonist-induced IP1 accumulation assays for HCA2/HCA3 chimeras 13
Table S2 | cAMP accumulation experiment for HCA1 mutants 14
Table S3 | Cryo-EM data collection, model refinement and validation statistics. 15

Figure S1 | The purification of 3,5-DHBA-HCA1, MK6892-HCA2, acifran-HCA2 and acifran-HCA3.

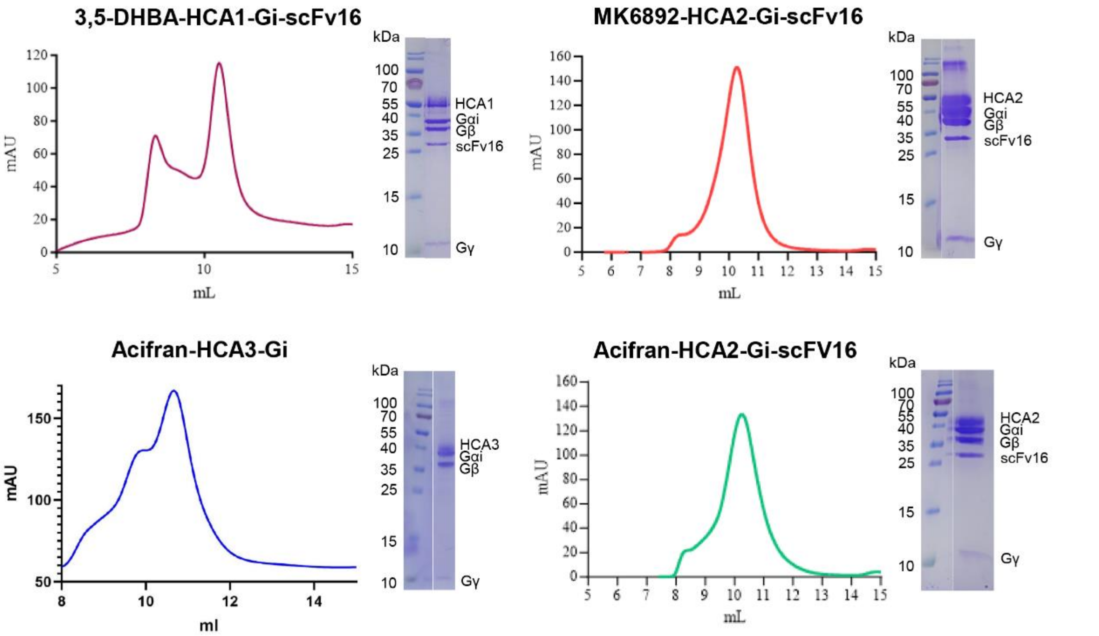

Representative elution profile and SDS-PAGE analysis of the 3,5-DHBA-HCA1, MK6892-HCA2, acifran-HCA2 and acifran-HCA3.

Figure S2 | Chemical structure of ligands of HCAs.

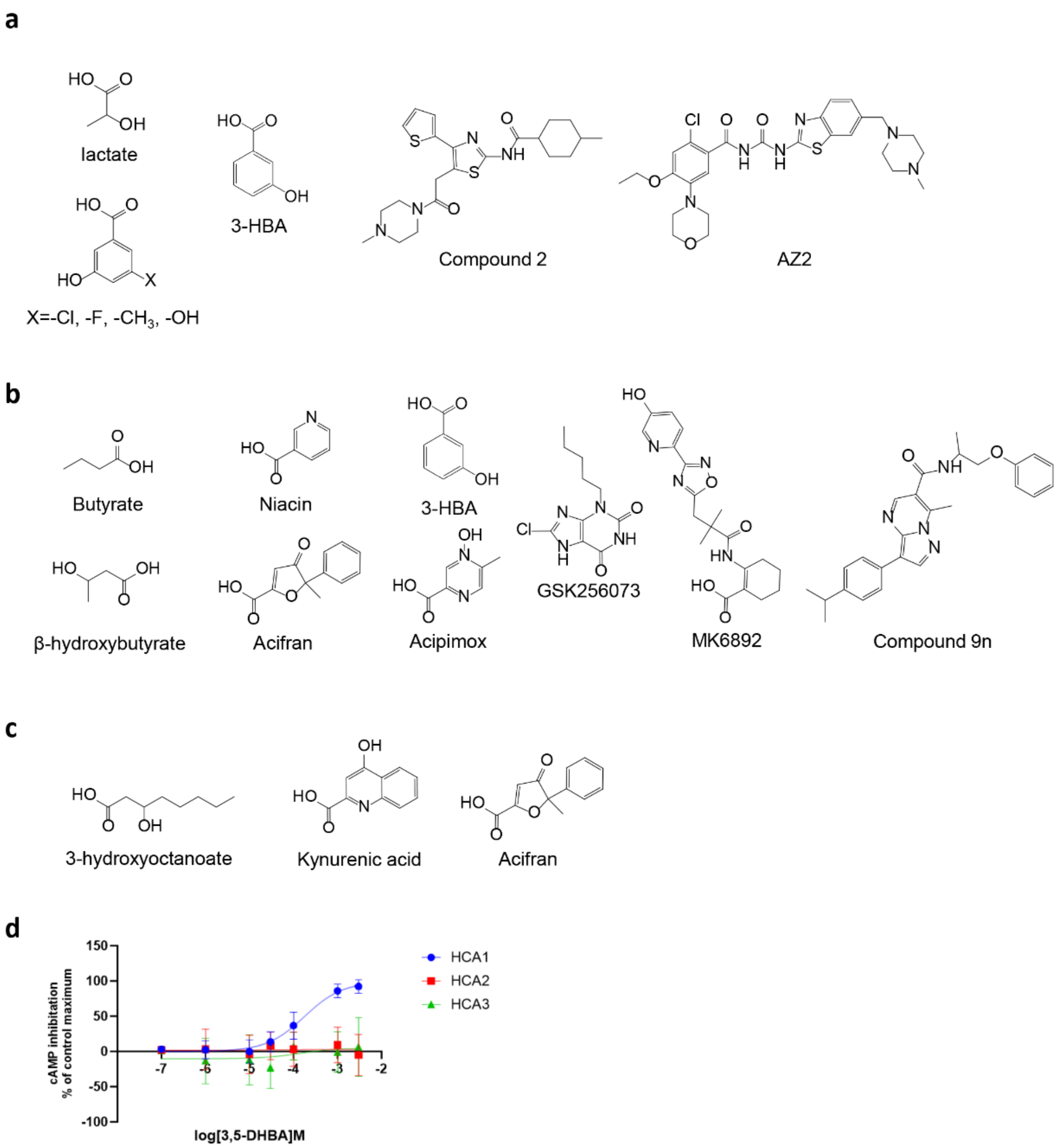

a-c. Chemical structures of typical ligands of HCA1(**a.**), HCA2(**b.**) and HCA3(**c.**) **d.** HCAs response curves induced by 3,5-DHBA. cAMP response was normalized to the wide type of HCA1. Data was shown as means ± SEM from three independent experiments, which were performed in triplicate.

Figure S3 | The cryo-EM data processing of 3,5-DHBA-HCA1, MK6892-HCA2, acifran-HCA2 and acifran-HCA3.

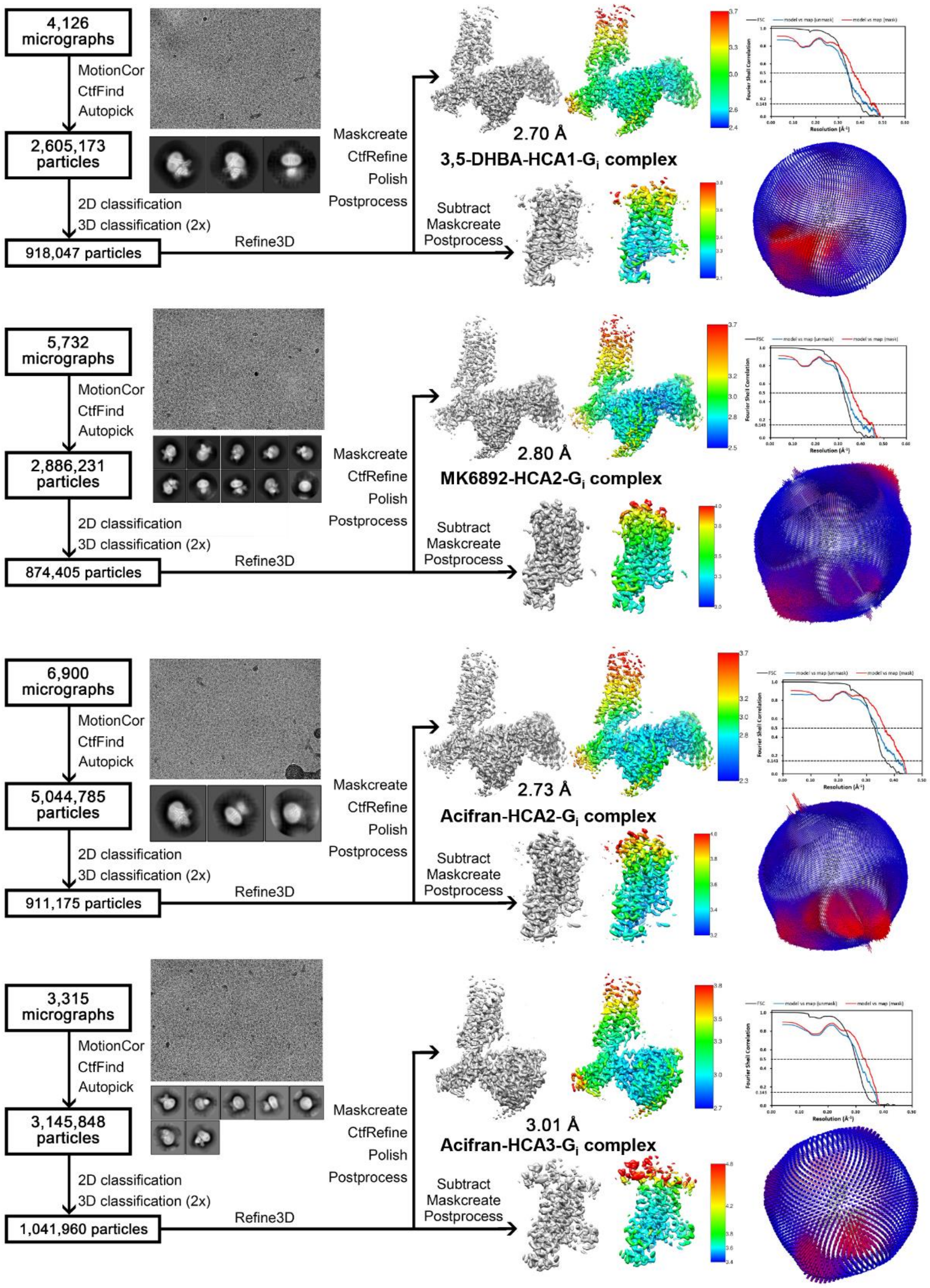

Cryo-EM micrographs and reference-free 2D class average of ligand-bound HCAs complex are shown in the flow chart of the cryo-EM data processing. The angle distribution and the FSC curves are shown in the right of this micrograph.

Figure S4 | Representative cryo-EM density of HCAs.

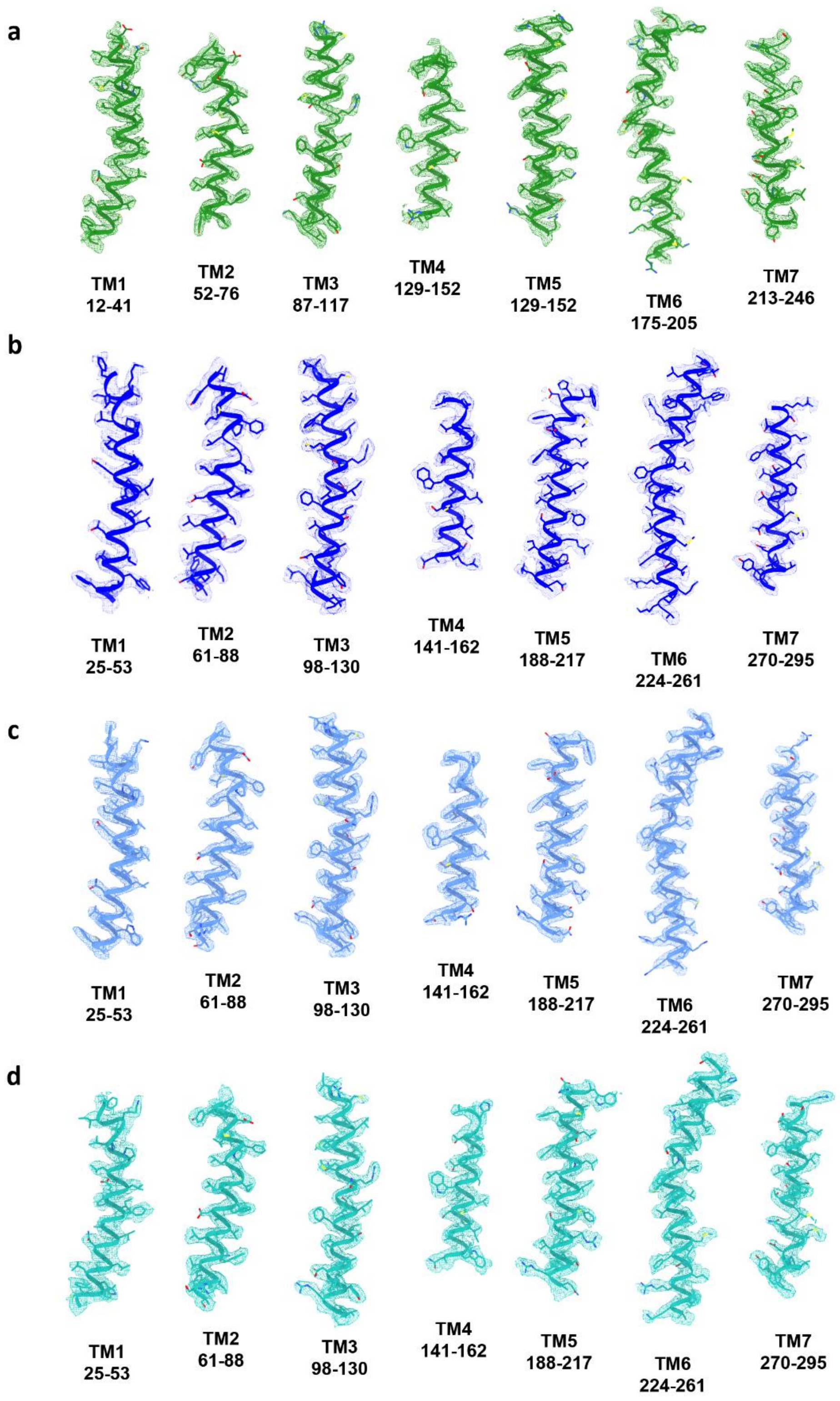

a. The transmembrane helix of 3,5-DHBA-HCA1. **b.** The transmembrane domain of MK6892-HCA2. **c.** The transmembrane domain of acifran-HCA2. **d.** The transmembrane domain of acifran-HCA3.

Figure S5 | The model of HCAs bound with agonists.

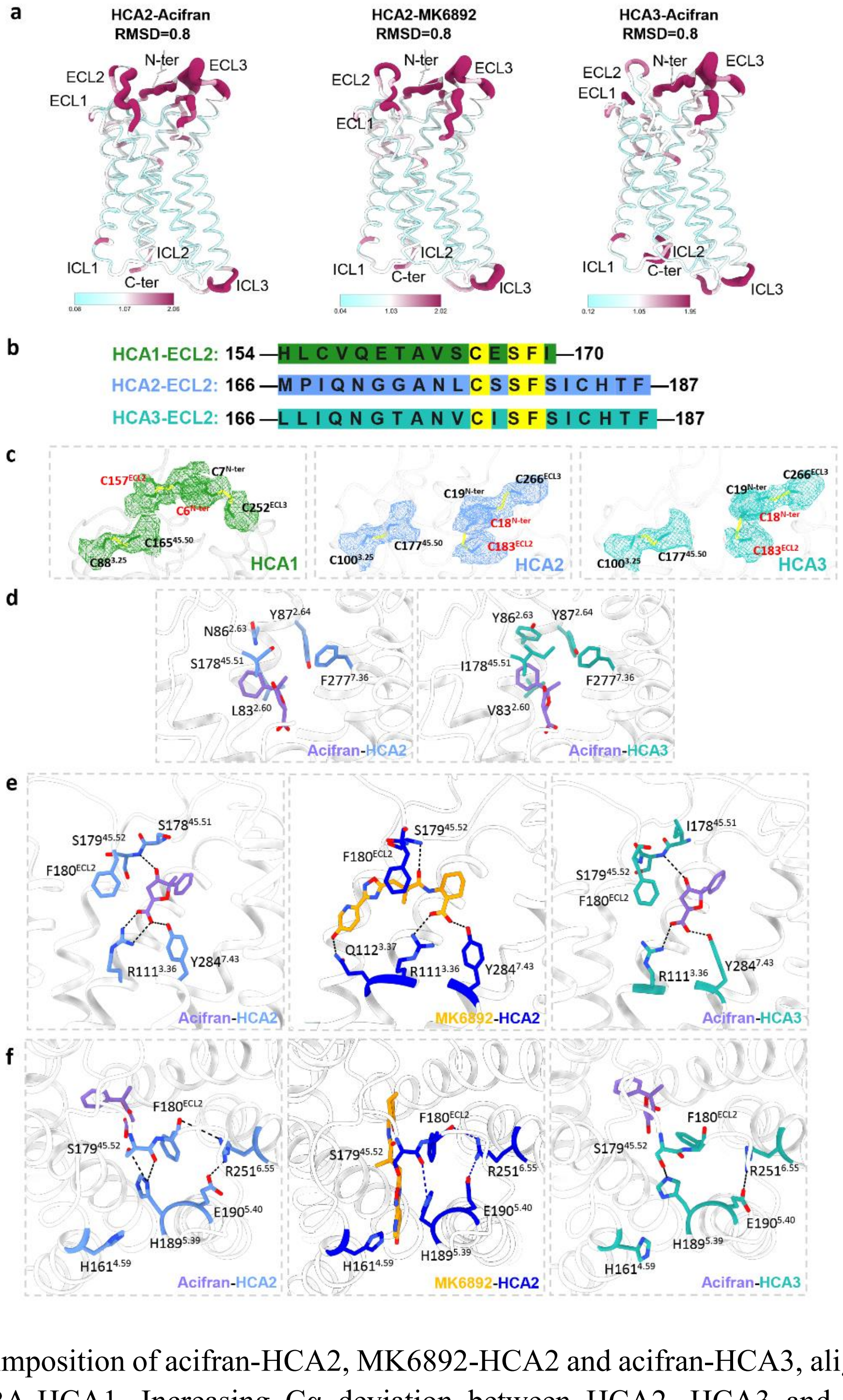

a. Superimposition of acifran-HCA2, MK6892-HCA2 and acifran-HCA3, aligned with 3,5-DHBA-HCA1. Increasing Cα deviation between HCA2, HCA3 and HCA1 is indicated by a color gradient from light blue to maroon. **b.** Sequence alignment of ECL2 in HCAs. The CxSF motif is highlighted with yellow block. HCA1 in forest green, HCA2 in medium slated blue, HCA3 in light sea green. **c.** Density map of three disulfide bonds, which is shown in mesh with a counter level of 0.003. **d.** The cavity formed between TM2, TM7 and ECL2. **e-f.** Detail interactions of the ligand-binding domain in HCA2 and HCA3, as well as the interactions above the ligand pocket.

Figure S6 | Molecule dock of lactate-HCA1 and 3-HBA with HCA2.

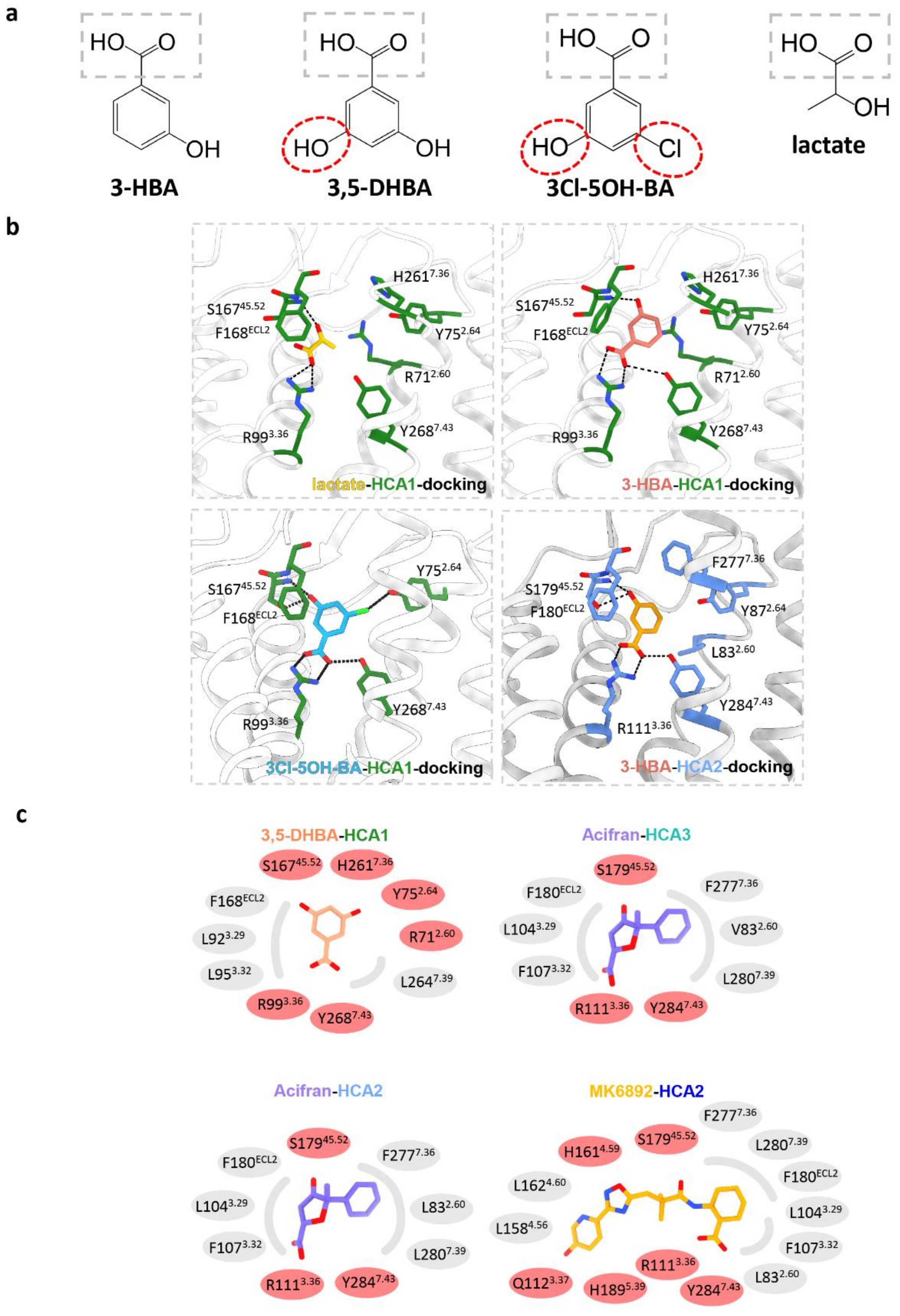

a. Chemical structures of 3-HBA, 3,5-DHBA, 3Cl-5OH-BA and lactate. The conserved groups are labeled in dotted rectangular colored in gray, the specific chemical structure shown in red circle colored in salmon. **b.** Detail interactions of major residues within 5 Å in the structure of molecule docking of lactate-HCA1, 3-HBA-HCA1, 3Cl-5OH-BA-HCA1 and 3-HBA-HCA2. **c.** The key residues in the ligand-binding pocket. Gray, hydrophobic residues. Salmon, polar residues.

Figure S7 | Sequence alignment of human HCA receptors.

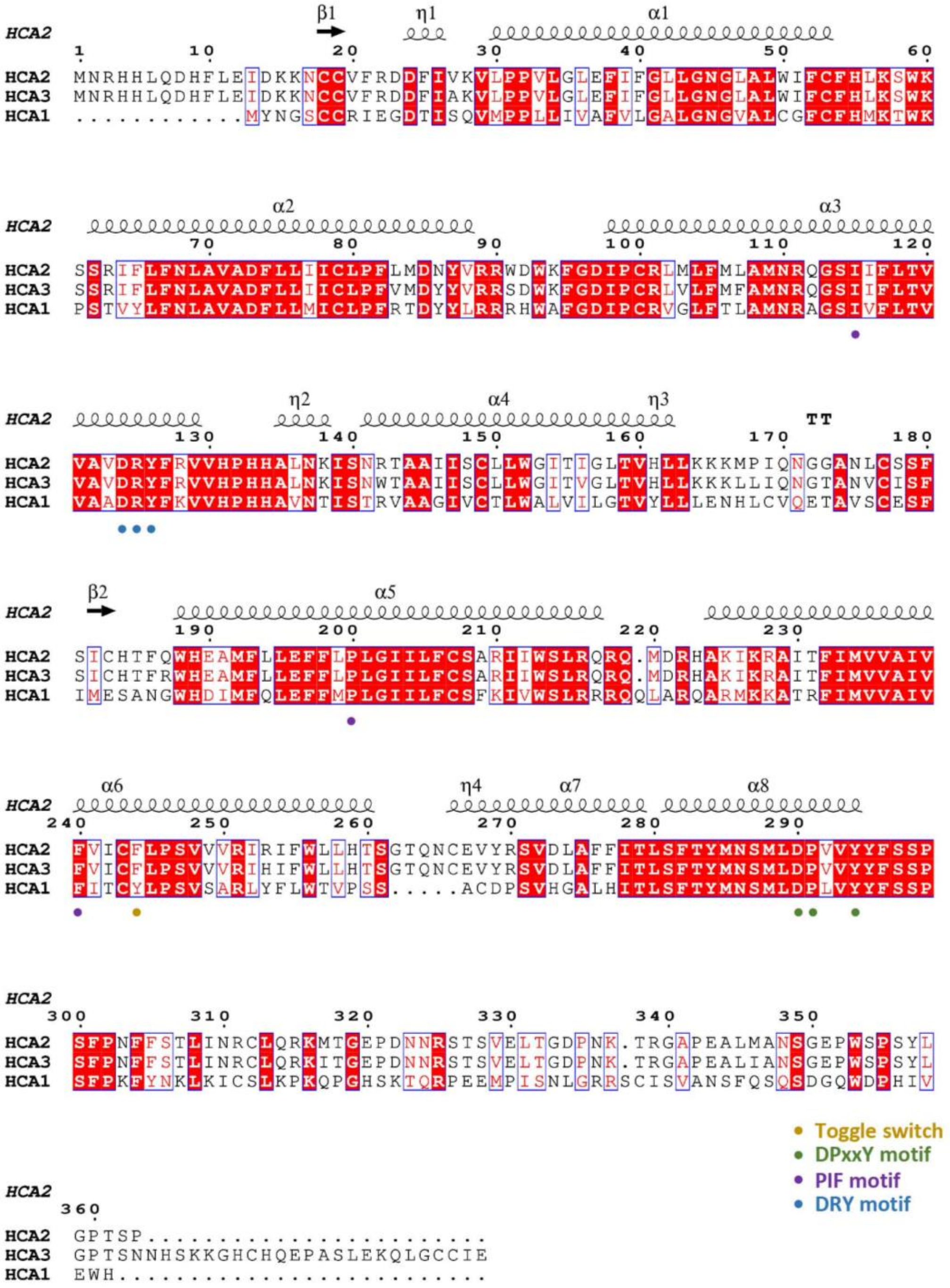

Residues including the conserved motifs (Toggle switch, DPxxY motif, PIF motif and DRY motif) are labeled by different colors dots.

Figure S8 | Activation of HCAs.

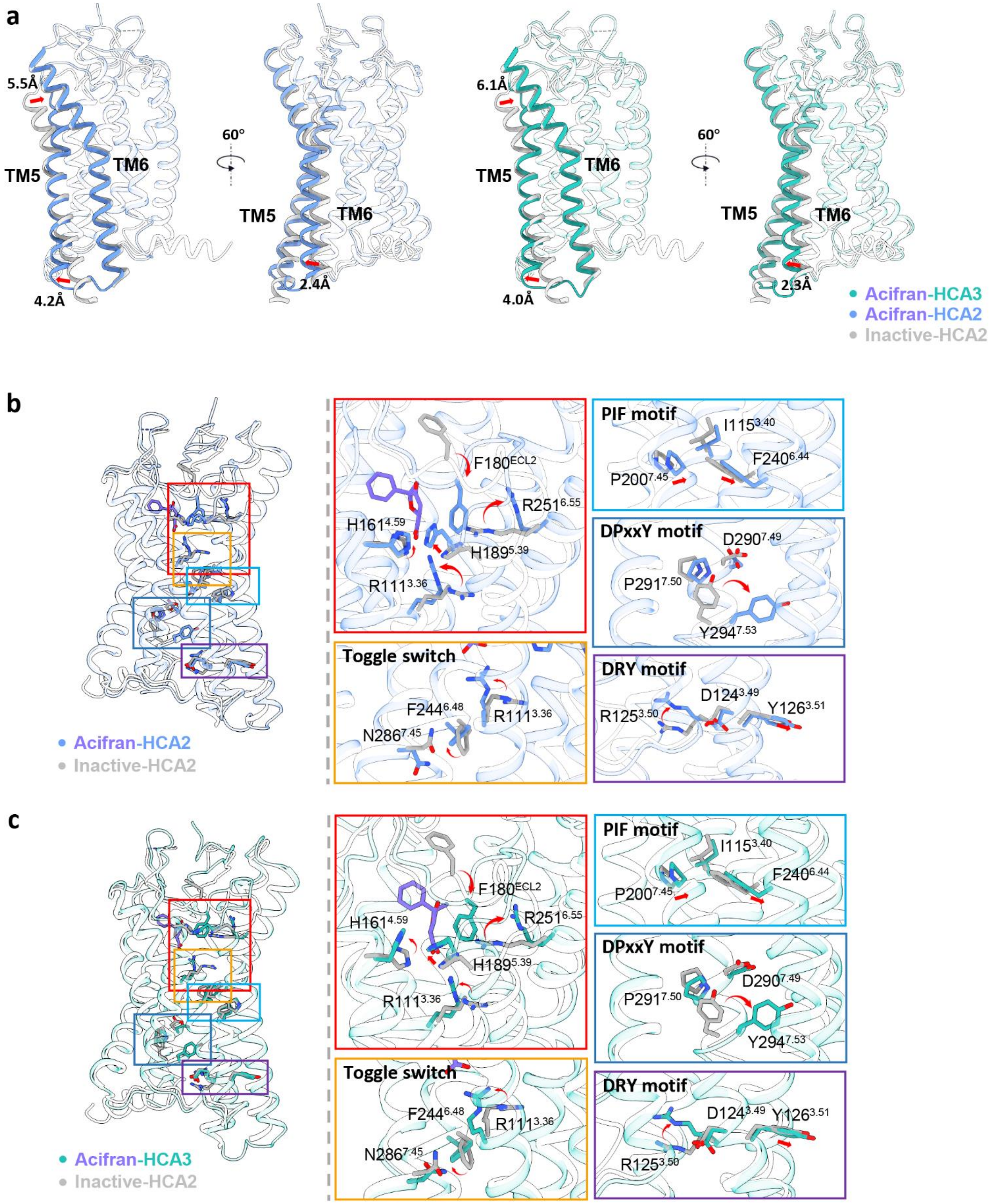

a. Superimposition of the active conformation of HCA2 (cornflower blue) and HCA3 (light sea green) with the inactive conformation of HCA2 (light gray, PDB: 7ZLY). **b-c.** Detail view of the rearrangement of the conserved residue $R99^{3.36}$ in **b.** HCA2 and **c.** HCA3, together with conserved motifs (toggle switch, PIF motif, DPxxY motif and DRY motif), compared with inactive state of HCA2.

Figure S9 | The interactions between HCA2/HCA3 and Gi.

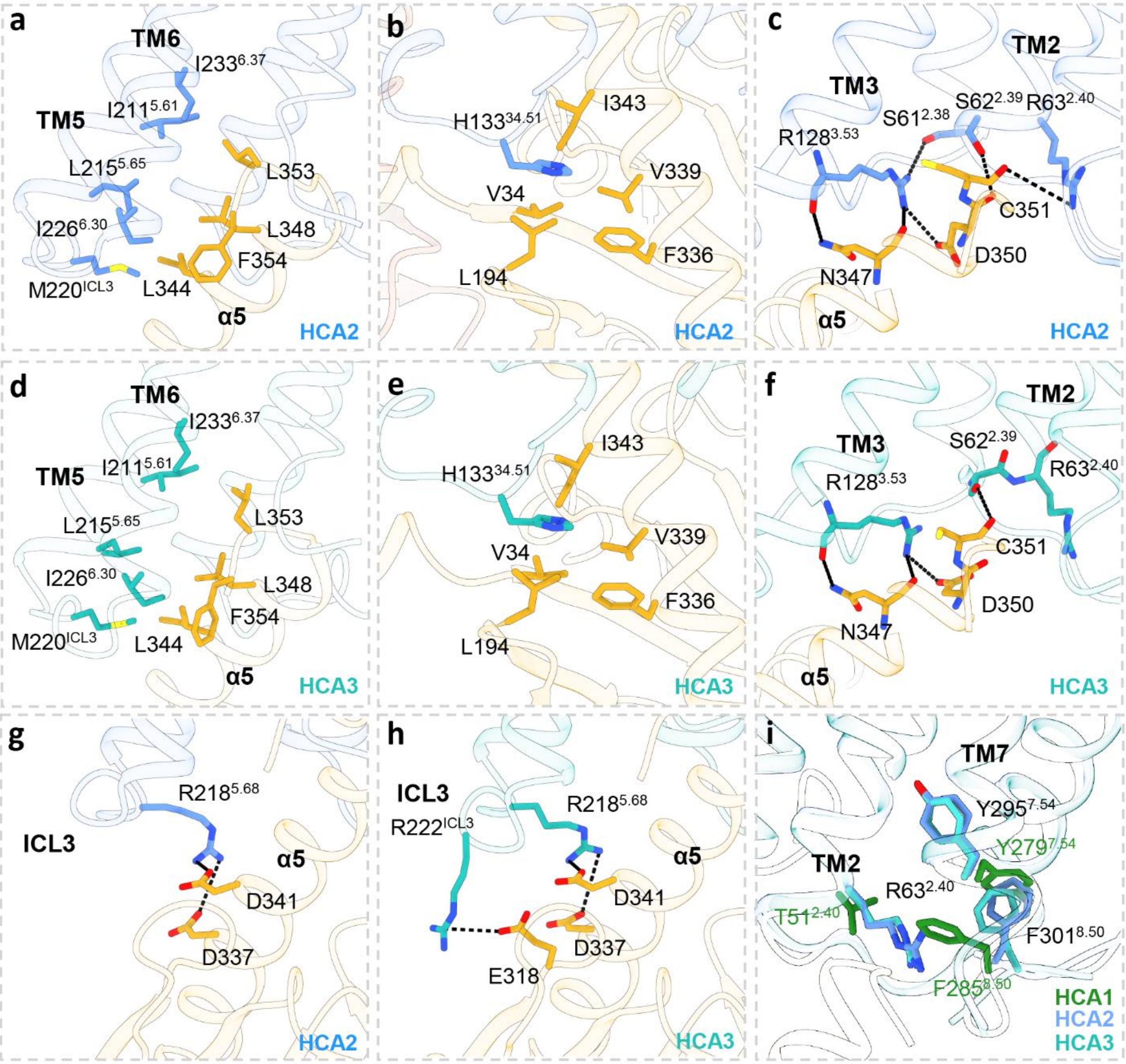

a. The hydrophobic interactions and the interlock network formed between HCA2 and α5 of Gαi. **b.** The hydrophobic interactions and the interlock network formed between HCA3 and α5 of Gαi. Black dotted line represents H-bonds and ionic interactions. Residues within 5 Å are labeled in these interactions and shown in stick representation. **c-d.** Detail connections formed by ICL2 in HCA2/HCA3 and α5 of Gαi. Black dotted line represents H-bonds and ionic interactions. Residues within 5 Å are labeled in these interactions and shown in stick representation. **e.** Structural comparison of T512.63 in HCA1 with arginine in HCA2/3, resulting the rotation of tyrosine in HCAs.

Figure S10 | Measurement of the cell surface expression level of mutant HCA1 constructs.

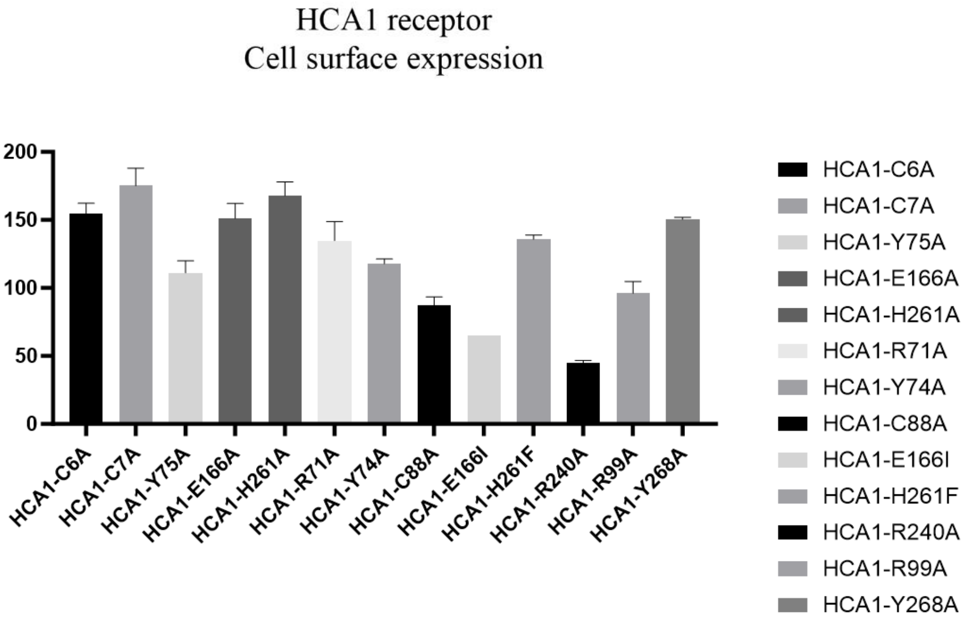

Data are mean ± SEM from 3~4 independent experiments (n=3~4).

Table S1 | Agonist-induced IP1 accumulation assays for HCA2/HCA3 chimeras

a.

Mutants	EC_{50} (nM)	Ratio	pEC_{50} ± SEM	Span (% of WT)	n	Expression (% of WT)
HCA2 WT	6.917	1.0	8.16±0.25	100±11	4	100
HCA2-HCA3(130-)	ND	ND	ND	ND	3	82±15
HCA3-HCA2(130-)	ND	ND	ND	ND	3	68±14
HCA2-HCA3(130-)+N86Y+W91S	ND	ND	ND	ND	3	76±21
HCA3-HCA2(130-)+S178I	ND	ND	ND	ND	3	129±17
HCA3-HCA2(130-)+Y86N+S91W	2.618	0.38	8.58±0.60	104±27	3	130±24
HCA2-HCA3(130-)+I178S	ND	ND	ND	ND	3	117±13
HCA3 WT	ND	ND	ND	ND	3	93±16

b.

Mutants	EC_{50} (μM)	Ratio	pEC_{50} ± SEM	Span (% of WT)	n	Expression (% of WT)
HCA3 WT	34.26	1.0	4.47±0.19	100±9	5	100
HCA2-HCA3(130-)	ND	ND	ND	ND	3	67±3
HCA3-HCA2(130-)	258	7.53	3.59±0.50*	130±41	3	79±22
HCA2-HCA3(130-)+N86Y+W91S	ND	ND	ND	ND	3	86±28
HCA3-HCA2(130-)+S178I	12.34	0.36	4.91±0.30	206±30***	3	138±15

a-b. EC_{50} values were determined after stimulation with increasing concentrations of (**a**) MK-6892/ (**b**) 3-Hydroxyoctanoic acid for 1 h at 37 ℃. Ratio is the percentage of EC_{50} to WT of the mutant. p EC_{50}±SEM shows the mean ± s.e.m. from at least three independent experiments performed in triplicate. Span is defined as the window between the maximum ligand reaction (Emax) and the carrier (no ligand). ND (not determined) refers to data where a robust concentration response curve could not be established within the concentration range tested. Number of experiments used for data processing is presented as n. Protein expression levels of chimeric complex on the cell surface are shown as percent compared to WT. * means ＜0.05 and *** represents as ＜0.001.

Table S2 | cAMP accumulation experiment for HCA1 mutants

a

Mutants	EC_{50} (μM)	pEC_{50} ± SEM	Span	n
HCA1 WT	193±40	3.7±0.1	100±1	3
HCA1 C6A	565±193	3.3±0.2	116±10	3
HCA1 C7A	657±352	3.3±0.2	94±16	3
HCA1 R71A	ND	ND	ND	3
HCA1 Y74A	ND	ND	ND	3
HCA1 Y75A	ND	ND	ND	3
HCA1 C88A	ND	ND	ND	3
HCA1 R99A	ND	ND	ND	3
HCA1 E166A	ND	ND	ND	3
HCA1 E166I	19±7	4.8±0.2	38±3	3
HCA1 R240A	35±29	4.8±0.4	51±8	3
HCA1 H261A	177±11	3.8±0.03	68±1	3
HCA1 H261F	23±11	4.7±0.2	27±2	3
HCA1 Y268A	ND	ND	ND	3
HCA2 WT	ND	ND	ND	3
HCA3 WT	ND	ND	ND	3

b

Mutants	EC_{50} (μM)	pEC_{50} ± SEM	Span	n
HCA1 WT	841±484	3.2±0.3	100±1	3
HCA1 R71A	ND	ND	ND	3
HCA1 Y74A	ND	ND	ND	3
HCA1 R240A	ND	ND	ND	3

EC_{50} values of HCA1 mutants and WT were determined after stimulation with increasing concentrations of 3,5-DHBA **(a.)** and lactate **(b.)**, while EC_{50} value of HCA2 WT and HCA3 WT also determined after activating by 3,5-DHBA **(a.)**. Ratio is the percentage of EC_{50} to WT of the mutant. p EC_{50}±SEM shows the mean ± s.e.m. from at least three independent experiments performed in triplicate. Span is defined as the window between the maximum ligand reaction (Emax) and the carrier (no ligand). ND (not determined) refers to data where a robust concentration response curve could not be established within the concentration range tested. Number of experiments used for data processing is presented as n. Protein expression levels of chimeric complex on the cell surface are shown as percent compared to WT.

Table S3 | Cryo-EM data collection, model refinement and validation statistics

	**3,5-DHBA-HCA1-Gi complex** (9KT9) (EMD-62560)	**MK6892-HCA2-Gi complex** (9KT7) (EMD-62558)
**Data collection and processing**		
Magnification	105,000	105,000
Voltage (kV)	300	300
Electron exposure ($e^-/Å^2$)	54	54
Defocus range (μm)	-1.0 ~ -1.5	-1.0 ~ -1.5
Pixel size (Å)	0.851	0.851
Symmetry imposed	C1	C1
Initial particle projections (no.)	2,605,173	2,886,231
Final particle projections (no.)	918,047	874,405
Map resolution (Å)	2.70	2.80
FSC threshold	0.143	0.143
Map resolution range (Å)	2.58 ~ 5.30	2.60 ~ 6.78
**Refinement**		
Initial model used	AlphaFold 2	9KT9
Model resolution (Å)	2.79	2.78
FSC threshold	0.5	0.5
Map sharpening B factor ($Å^2$)	-102.6	-61.9
Model composition		
Non-hydrogen atoms	8,951	9,094
Protein residues	1,142	1,150
Ligand	1	1
B-factors ($Å^2$)		
Protein	6.03/141.00/54.72	9.37/154.73/65.78
Ligand	121.37/121.37/121.37	123.01/123.01/123.01
R.m.s. deviations		
Bond lengths (Å)	0.003	0.003
Bond angles (°)	0.600	0.600
Validation		
MolProbity score	1.43	1.50
Clashscore	4.45	5.26
Rotamer outliers (%)	0.00	0.00
Ramachandran plot		
Favored (%)	96.72	96.57
Allowed (%)	3.28	3.43
Disallowed (%)	0.00	0.00

	Acifran-HCA2-Gi complex (9KT8) (EMD-62559)	Acifran-HCA3-Gi complex (9KT6) (EMD-62557)
**Data collection and processing**		
Magnification	105,000	105,000
Voltage (kV)	300	300
Electron exposure ($e^-/Å^2$)	54	54
Defocus range (μm)	-1.0 ~ -1.5	-1.0 ~ -1.5
Pixel size (Å)	0.851	0.851
Symmetry imposed	C1	C1
Initial particle projections (no.)	5,044,785	3,145,848
Final particle projections (no.)	911,175	1,041,960
Map resolution (Å)	2.73	3.01
FSC threshold	0.143	0.143
Map resolution range (Å)	2.56 ~ 6.36	2.83 ~ 7.36
**Refinement**		
Initial model used	9KT9	9KT9
Model resolution (Å)	2.75	2.99
FSC threshold	0.5	0.5
Map sharpening B factor ($Å^2$)	-69.1	-89.1
Model composition		
Non-hydrogen atoms	9,082	7,263
Protein residues	1,150	914
Ligand	1	1
B-factors ($Å^2$)		
Protein	6.09/173.53/65.27	7.52/163.87/65.79
Ligand	134.50/134.50/134.50	122.66/122.66/122.66
R.m.s. deviations		
Bond lengths (Å)	0.002	0.002
Bond angles (°)	0.563	0.655
Validation		
MolProbity score	1.39	1.59
Clashscore	4.60	4.65
Rotamer outliers (%)	0.00	0.00
Ramachandran plot		
Favored (%)	97.10	95.35
Allowed (%)	2.90	4.65
Disallowed (%)	0.00	0.00
